# Changing Primary School Children’s Engagement in Active School Travel Using Safe Routes to School Interventions: A Rapid Realist Review

**DOI:** 10.3390/ijerph19169976

**Published:** 2022-08-12

**Authors:** Thomas V. Vasey, Suzanne J. Carroll, Mark Daniel, Margaret Cargo

**Affiliations:** Health Research Institute, University of Canberra, Building 23, 11 Kirinari St., Bruce, ACT 2617, Australia

**Keywords:** Active School Travel, active school transportation, Safe Routes to School, interventions, physical activity, children, Rapid Realist Review

## Abstract

Safe Routes to School (SR2S) interventions have been implemented in many economically developed countries to improve children’s engagement in Active School Travel (AST). Evaluations have highlighted inconsistencies in SR2S intervention outcomes, raising questions as to how, why, and under what contextual conditions these interventions work. This review used a Rapid Realist Review (RRR) methodology to build, test, and refine an overarching program theory that unpicks the contextual factors and underlying mechanisms influencing children’s engagement in AST. From the 45 included documents, 16 refined Context–Mechanism–Outcome Configurations (CMOCs) were developed and clustered into three partial program theories (i.e., implementor/implementation, child, and parent), with the associated mechanisms of: (1) School Reliance; (2) School Priority; (3) Fun; (4) Pride; (5) Perceived Safety; (6) Distrust; (7) Convenience; (8) Perceived Capabilities; and (9) Reassurance. The overarching program theory delineates the pathways between intervention implementation, children’s motivation, parental decision-making, and children’s engagement in AST. The findings suggest SR2S interventions can motivate children to engage in AST, but whether this motivation is translated into engagement is determined by parental decision-making. This review is novel for highlighting that many of the factors influencing parental decision-making are contextually driven and appear to be unaddressed by the current suite of SR2S intervention strategies. The review additionally highlights the complexity of parental perceptions of safety, with the traffic and the road environment shaping only part of this multidimensional mechanism. Practitioners and policymakers need to tailor SR2S interventions to local contexts to better influence parental decision-making for children’s engagement in AST.

## 1. Introduction

Physical activity is an important behaviour for children’s health. It is associated with a broad range of health benefits, including improvements to cardiorespiratory fitness, cardiometabolic health, cognitive outcomes (e.g., academic performance), mental health, and reduced adiposity [1]. It is recommended that children aged 5–17 years engage in 60 min of daily moderate to vigorous physical activity, as well as muscle and bone strengthening activities three times per week [2]. An analysis of physical activity report cards from across 49 countries found that approximately 70 percent of children do not meet this recommendation [3].

Active School Travel (AST), commonly defined as any form of human-powered transportation to and/or from school (e.g., walking, cycling, scootering, or skating), has been recognised as a promising strategy for increasing children’s habitual engagement in physical activity. Evidence supports a positive association between AST and children’s daily moderate to vigorous physical activity [4,5], improvements in cardiovascular fitness [6], and body composition [7].

Despite growing evidence supporting children’s engagement in AST, many economically developed countries have experienced significant declines in AST [8,9]. For example, van der Ploeg et al. analysed household travel surveys in New South Wales, Australia at four time points between 1971 and 2003 and reported a 32.2 percent decline in children’s engagement in AST [10]. The reasons for this decline are complex, with poor walkability and street connectivity of neighbourhoods [11], parental time constraints and convenience of passive travel [11,12], and parental perceptions of neighbourhood safety [11,12] frequently cited barriers.

To mitigate these barriers, AST interventions including Safe Routes to School (SR2S), the “walking school bus”, school travel planning, crossing supervisors, road and bicycle education, incentive schemes, and gamification have been implemented [13,14,15]. Of these interventions, SR2S has received substantial financial investment in the United States [16], leading to growing interest in Australia, Canada, New Zealand, Sweden, and the United Kingdom. SR2S interventions aim to promote children’s use of active modes of travel to and/or from school by encouraging participation and improving the safety of the environment surrounding schools [17]. To achieve this, SR2S interventions apply a set of complementary strategies guided by the Six E’s framework [18]: Education, Encouragement, Engineering, Engagement, Equity, and Evaluation. Education strategies provide children and the broader community with the knowledge and skills to actively travel safely (e.g., SR2S maps to enable children to navigate a safe route to school). Encouragement strategies create enthusiasm and motivate children to choose active travel (e.g., incentives such as activity vouchers for reaching travel milestones). Engineering strategies involve structural changes to the built environment to make active travel safer and more convenient (e.g., installation of a new footpath). Engagement strategies ensure that the voices of various stakeholders (e.g., children, parents, teachers, and/or community organisations) are acknowledged and used to inform intervention planning and implementation (e.g., child walk-along interviews to gain children’s perspectives of AST). Equity strategies ensure that SR2S interventions are designed to benefit children from all backgrounds (e.g., children with disabilities). Evaluation assesses whether the intervention has been successful and why/why not to improve future approaches (e.g., was there an impact on children’s engagement in AST).

To date, there has been no systematic review of only SR2S interventions. However, existing reviews of AST interventions that include SR2S interventions highlight the mixed intervention results that SR2S interventions have achieved on children’s engagement in AST [13,14,15,19,20,21]. Inconsistencies in intervention outcomes raise questions as to how and why these interventions have worked or not, and under what contextual conditions these interventions work. Answering these questions is important given calls for AST interventions, such as SR2S, to transition away from a ‘one size fits all’ approach to implementation in favour of context-specific approaches that consider a school’s local context, identified needs, priorities, and level of resourcing [22]. The multi-component and flexible nature of SR2S interventions requires an approach to evidence synthesis that can capture the complexity of implementation and its impacts on children’s engagement in AST.

To address the need for a review sensitive to the contextual complexity of children’s engagement in AST and SR2S intervention implementation, a theory-driven realist review approach was adopted for this study, guided by the question: How, why, and under what circumstances do SR2S interventions impact primary school children’s engagement in AST in high-income countries? More specifically; (1) What are the contextual factors relevant to children’s engagement in AST at the child, parent, family/household, neighbourhood, and school levels? and (2) What are the underlying mechanisms associated with children’s engagement in AST, as experienced by children, parents, or adults engaged in intervention implementation? Review findings will be used to generate recommendations to inform the development and implementation of SR2S interventions in Australia and other high-income countries.

## 2. Materials and Methods

### 2.1. Methodology

Realist methodology is a theory-driven approach to evidence synthesis that goes beyond whether an intervention simply works, by seeking to explain “what works, for whom, in what respects, to what extent, in what context and how?” [23,24,25]. It does this by making transparent the underlying assumptions for how interventions work or not, known as program theory [23,24]. Program theory takes the form of a number of Context–Mechanism–Outcome Configurations (CMOCs) that explain how an intervention strategy (e.g., what the intervention offers participants), when introduced into specific *contexts* (e.g., features of the existing settings), activates or deactivates one or more *mechanisms* (e.g., if and how the ‘reasoning’ of an actor changes), to generate an *outcome* (i.e., intended or unintended result) [23,24]. In other words, mechanisms are defined by the resources offered by intervention strategies and the response of actors to these resources. In realist synthesis, reviewers begin with the formulation of preliminary program theory (i.e., preliminary CMOCs), that explain how, why, and in what contexts an intervention is deemed to work or not. These preliminary CMOCs are then tested using existing literature to either confirm, refute, or refine the CMOCs, develop emergent CMOCs, and refine the overarching program theory. The realist model of causation is generative, meaning that the manifest world is viewed as being generated or caused by underlying mechanisms [24]. The logic of inquiry is retroductive and aims to uncover hidden mechanisms that are embedded within the social system. Unlike traditional systematic reviews (e.g., meta-analyses), intervention effectiveness is not determined on the basis of average effect sizes by pooling data across primary studies. Average effect sizes may obscure explanations about causative factors, which are key to understanding realist causation [23]. Realist analysis seeks explanation through highly nuanced context-dependent configurations (i.e., CMOCs) rather than generalised main effects. As such, refined program theory is used to inform realist evaluations and policy decision making for intervention implementation [23,24,25].

#### Rapid Realist Review

This review used a specific type of realist review known as a Rapid Realist Review (RRR). RRR methodology was put forth by Saul et al. as a time efficient alternative to a full realist review that can generate knowledge to inform policy and implementation decision-making under time constraints [26]. RRR was deemed the most suitable approach as it aligned with the pragmatic need to develop and refine program theory to guide the realist evaluation of SR2S interventions in two Australian states.

The Realist and Meta-narrative Evidence Synthesis: Evolving Standards (RAMESES) are internationally recognised publication standards for realist synthesis [27]. Developed using a Delphi method with an interdisciplinary panel of experts in evidence synthesis and realist research, these publication standards outline structural and content requirements for prospective realist synthesis publications to strengthen reporting and quality assessment. Key components of the realist synthesis method as described in the RAMESES [27] are: (1) an initial scoping of a broad range of literature and development of preliminary program theories (i.e., preliminary CMOCs) based on diverse data sources, including input from experts and stakeholders (see Section 2.2 and Section 2.3); (2) the identification and selection of theoretically relevant evidence for inclusion in the synthesis (see Section 2.4 and Section 2.5); and (3) the testing and refinement of the preliminary program theories using the selected theoretically relevant evidence (see Section 2.6 and Section 2.7). This review adhered to the explanatory guidance outlined in the RAMESES publication standards for realist synthesis [27], and was informed by Saul et al.’s protocol for conducting a RRR [26].

### 2.2. Advisory Panels

Per the RRR protocol, expert advisory and local reference panels were formed to assist with the review process. Similar to that of Brown et al. [28], this review also formed part of the lead author’s Ph.D. thesis. As such, the expert advisory panel (*n* = 3) was comprised of the lead author’s Ph.D. supervisory panel, who provided expertise in the areas of children’s AST, the built environment, and realist methodology. The role of the expert advisory panel was to provide methodological guidance such as refining the review scope, streamlining the literature search process, and/or assisting with the synthesis and interpretation of the review’s findings [26]. The local reference panel (*n* = 4) was comprised of an industry expert with a background in implementing AST interventions and parents with an understanding of the primary school context. The local reference panel’s role was to validate the applicability and usability of the review’s findings and to ensure they reflect real-world experience and current practice [26]. Where applicable, the specific involvement of each panel in this review has been described under the following subheadings.

### 2.3. Literature Scoping

A scoping exercise was conducted by the lead reviewer between October 2019 and February 2020 to develop an understanding of the topic area, gauge the feasibility and scope of the review, and develop the review’s preliminary CMOCs. The scoping exercise involved reviewing diverse data sources including published research articles, grey literature reports, news articles, and websites. The preliminary CMOCs were formulated using a combination of the lead reviewer’s knowledge of the topic, the important contextual factors, mechanisms, and outcomes identified during the informal data source scoping, and ongoing consultation with the expert advisory panel who provided guidance, supervision, and strategic input for the development of the preliminary CMOCs.

**Figure 1:**
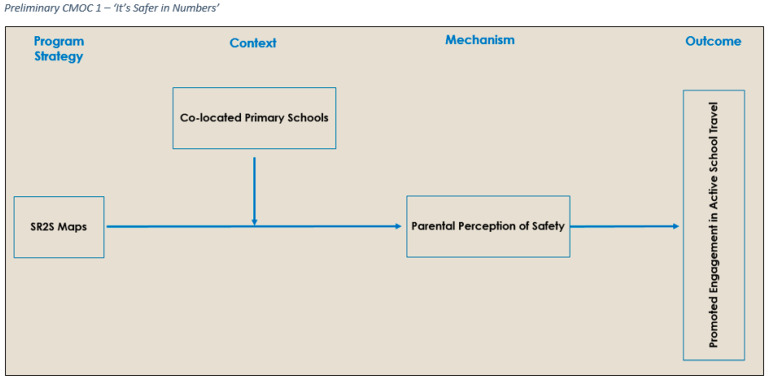
Example Preliminary CMOC.

From the scoping exercise, 13 preliminary CMOCs were developed, theorising how, why, and under what circumstances SR2S interventions were deemed to work or not. The 13 preliminary CMOCs were then presented and discussed with the review’s local reference panel to aid with sense-checking and further refinement. This step strengthened the plausibility of the review’s initial theoretical formulation. During the discussion, the local reference panel raised the absence of preliminary CMOCs for children and implementors. Following the meeting, an additional three preliminary CMOCs (two child/one implementation) were developed. The 16 preliminary CMOCs were organised to represent three partial program theories that corresponded to the domains of Implementors/Implementation, Child, and Parent and were used as the theoretical basis guiding the analysis. An example preliminary CMOC, relating to the parent partial program theory, can be seen in Figure 1. To illustrate, when SR2S maps (a key strategy of SR2S interventions) are introduced into two co-located primary schools (C), the greater number of active travellers using the same routes to get to school will change parental perception of safety (M), leading to children’s promoted engagement in AST (O). The remaining preliminary CMOCs from each category can be found in Supplementary File S1.

### 2.4. Literature Searching and Screening

In realist synthesis, literature searching is an inclusive process that acknowledges that data relevant for testing aspects of the preliminary CMOCs can lie in a broad range of sources that, for example, may cross discipline, programmatic or domain boundaries [27]. Realist synthesis can include published evaluations, unpublished evaluation reports, commentaries, non-intervention research, author interpretations, and theoretical papers, and treats them on fairly equitable grounds [23]. Practically, a synthesis starts with gathering evidence that is most specific to the review question, and, as part of the theory testing and refinement process, the search is broadened to supplement this literature with theoretically relevant information sources. The inclusion of documents is driven by their theoretical relevance since the aim of a realist synthesis is to develop middle range theory—theory that is sufficiently specific to generate propositions that can be tested about aspects of SR2S interventions, but abstract enough to be applicable to other AST interventions. To account for this, two searches of the APA PsycInfo, CINAHL, Scopus, and Web of Science bibliographic databases were conducted during March 2020. The first search identified literature specific to SR2S interventions. The second search, conducted five days later, identified broader literature relevant for theory development, testing and/or refinement (e.g., other AST intervention/non-intervention).

Search terms for the first search were informed by existing systematic reviews for AST [13,14,15,19] and refined in consultation with the expert advisory panel and the review’s research librarian. Following the research librarian’s advice, and preliminary database searches, a text string intended to capture specific methods in searches (i.e., methods string) was deemed too restrictive to be included. Table 1 illustrates the search strategy used for the Scopus database. The full search strategy for the first search can be seen in Supplementary File S2.

Similar to the first search, search terms for the second search were informed by existing AST systematic reviews in consultation with the expert advisory panel and the review’s research librarian. The second search was used to identify broader literature in support of the contextual factors and associated mechanisms influencing children’s engagement in AST. This focus was informed by the scoping exercise that illuminated a ‘black box’ understanding of how these interventions worked. As qualitative approaches are more commonly used to explore contextual factors and mechanisms in realist research [29], a qualitative methods’ search string was included in the search strategy. Search terms for the qualitative methods string were informed by the Cochrane Qualitative and Implementation Methods Group [30]. The search strategy for the second search can be seen in Supplementary File S2.

Search strings for both searches were adapted accordingly for each database. No geographic or date limits were set. Due to COVID-19 related delays, both searches were updated in September 2021. All grey literature documents were identified through the expert advisory panel.

For the first search, documents were included if they: (1) involved SR2S interventions (defined as an intervention that promoted ‘safe routes’ and was informed by components of the 6 E’s framework [18]); (2) were conducted in a high-income country (as listed by The World Bank [31]); (3) targeted children of primary school age (i.e., 5 to 12 years of age), or a sub-group of these ages (e.g., 9 to 10 years of age); and (4) were written in English. For the second search, documents were included if they: (1) involved any type of AST intervention or the topic of study involved active travel, independent play, independent physical activity, and/or independent mobility; (2) were conducted in a high-income country; (3) targeted children of primary school age (i.e., 5 to 12 years of age), or a sub-group of these ages (e.g., 9 to 10 years of age); (4) were qualitative or mixed method, providing qualitative results were reported separately; and (5) were written in English.

For each search, a two-stage screening process was performed by the lead reviewer with the assistance of a second reviewer, using inclusion criteria developed by the review team. In the first stage, titles and abstracts from the searches were screened. In the second stage, full-text documents were retrieved and reviewed to evaluate whether the review’s inclusion criteria were met. To ensure the inclusion criteria were applied consistently throughout the screening process, a 10% random sample of eligible documents at the first screening stage (i.e., title and abstract) and second screening stage (i.e., full text) were screened independently by a second reviewer. Inter-rater reliability was calculated using Cohen’s Kappa Coefficient. There was almost perfect agreement between the two reviewers at stage 1 (κ = 0.897, 95% CI, 0.832 to 0.961) and substantial to almost perfect agreement between reviewers at stage 2 (κ = 0.900, 95% CI, 0.709 to 1.000). Where disagreements between reviewers arose, they were discussed and resolved. In the three instances where disagreement could not be resolved, a third reviewer acted as an arbitrator.

### 2.5. Document Selection and Appraisal

Upon completion of the full text screening, documents were assigned to categories (i.e., SR2S, other AST intervention, and non-intervention) ready for selection. Document selection in realist synthesis requires documents be judged and selected according to the realist principles of relevance (i.e., whether they can contribute to theory building and/or testing) and rigour (i.e., whether the methods used to generate a given observation or inference were credible and trustworthy) [32]. As this review focused on understanding how, why, and under what circumstances SR2S interventions impact school-aged children’s engagement in AST, documents pertaining to SR2S interventions were prioritised for inclusion. Documents from remaining categories were sampled to provide further support for testing and refinement of the CMOCs and overarching program theory.

Similar to the approach of Price et al. [33], a quality appraisal of the included documents was not conducted, given that appraisal tools cannot sufficiently capture the different ways documents can contribute to theory development and testing, such as valuable ‘nuggets’ of data being contained within studies of limited quality [32].

### 2.6. Data Extraction

Descriptive data for the included documents were extracted using a tool developed and piloted by the review team. The lead reviewer extracted details of author(s), year of publication, intervention type, study aim/purpose, methodology, guiding theory, model or framework, location of study, study setting, participants, socio-demographic factors, data collection methods, analytic approach, and a summary of the main findings.

In realist synthesis, the purpose of data extraction is to support the analysis and synthesis process. To do this, data are extracted according to realist logic; that is, data are coded/extracted to reflect contextual factors, mechanisms, and outcomes [27]. In this review, the data extraction was informed by a social-ecological approach [34]. Context codes were tabulated into their corresponding category (e.g., child, parent, family/household, neighbourhood, school, implementation, and other), along with coded mechanisms, outcomes, and supporting quotations and data extracts (i.e., descriptive information from the primary text). To refine the extraction tool and cross-check interpretations of CMOCs, the lead reviewer and a second reviewer (TVV/MC) independently coded and discussed five articles.

In the first step of the CMOC analysis, all documents were read a minimum of five times and coded using a colour-coded system for data sections that represented context, mechanism, and outcome. There was an apparent scarcity of context within the included quantitative SR2S evaluation documents. This lack of context was likely the result of site-level variations being lost during data aggregation due to the large-scale nature of the SR2S evaluations. In the second step, coded data were transferred into a coding matrix developed in Microsoft Excel v16 [35], which provided transparency and an audit trail for the coding process. Primary documents were hyperlinked to the Excel coding matrix and an additional column was added to allow the lead author to record notes that facilitated the theory building and/or refinement process.

### 2.7. Data Analysis and Synthesis

The synthesis was undertaken by the lead author using a realist logic of analysis [24,36]. The logic of enquiry in realist analysis is *configurational*, and sees the iterative testing and refinement of initial explanatory theories (i.e., preliminary CMOCs) detailing how program strategies, when introduced into existing contexts, activate or deactivate mechanisms, leading to an outcome [24]. This is done using a diverse range of empirical data sources [27]. In this review, the 16 preliminary CMOCs contained within the three partial program theories were used as the theoretical basis of analysis, and as a guide for identifying patterns in the coded data, including the relationships between the partial program theories. The coding matrix in Excel was searched to identify patterns in the contextual conditions (e.g., high levels of neighbourhood crime) and SR2S intervention strategies (e.g., SR2S maps) under which mechanisms (e.g., parental perceived safety of stranger danger) were present or absent. In many cases, the patterns identified in the coded data provided only partial components of the CMOCs (e.g., context and outcome only). Using Pawson’s Reasoning Processes of adjudicating, consolidating, juxtaposing, reconciling, refining, and situating [24], the identified patterns were then used to build CMOCs that either confirmed, refuted, or refined the preliminary CMOCs (i.e., testing) or provided sufficient support for the development of emergent CMOCs. For example, in one study, an AST intervention was introduced into a school neighbourhood that had unfavourable topography (C). Unfavourable topography, whether objectively measured or subjectively perceived (e.g., by parents), is considered an aspect of context (i.e., a feature of the existing setting). In this study, unfavourable topography detracted from children’s engagement in AST (O). However, the explanation (M) was absent. In a second study, with a similar topography, perceived child capabilities (M) were discussed as being a barrier to children’s engagement in AST. These findings were then superimposed to build and/or refine the CMOC. The CMOCs were refined within each partial program theory and in relation to the other partial program theories to build and refine an overarching program theory that provided a holistic understanding of how implementation, child, and parent factors interacted together to influence children’s engagement in AST. This was an iterative process that required continuous movement between the coded data, the synthesised CMOCs, overarching program theory, and consultations with the expert advisory panel. All CMOCs were synthesised at a middle range, such that they were specific enough to be tested and refined but abstract enough to apply to other AST interventions. Prior to finalising the synthesis, all refined CMOCs and the overarching program theory were presented to the local reference panel for further sense-checking and refinement.

## 3. Results

### 3.1. Document Characteristics

**Figure 2:**
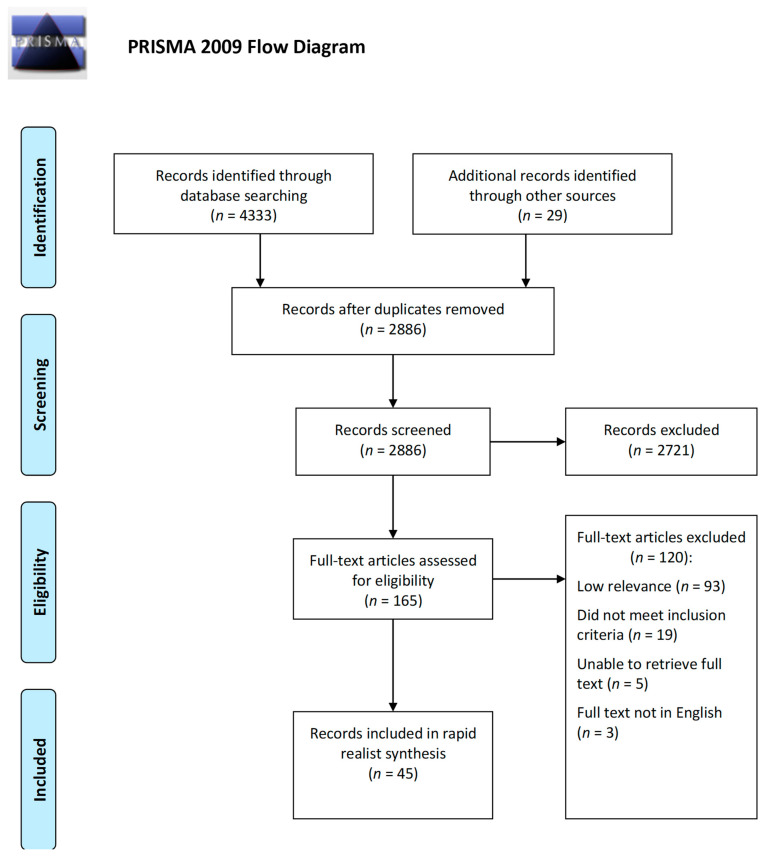
Adapted PRISMA Flow Diagram for RRR.

This RRR included 45 documents following the screening of 2886 (Figure 2). As shown in Table 2, just over half of the included documents originated from the US (51.1%). The majority of the included documents were peer-reviewed research articles (84.4%) and used qualitative methodologies (55.6%). Twenty of the included documents (44.4%) were SR2S interventions, with two of these (4.4%) combining SR2S with another AST intervention (e.g., SR2S combined with a walking school bus). Sixteen of the included documents (35.6%) were not associated with an intervention but were deemed highly relevant for theory testing and refinement.

Relating document characteristics to the analysis process, the preliminary CMOCs were tested and refined first by examining the SR2S documents, and then by sampling AST intervention types relevant to the six SR2S intervention strategies. For example, in walking school bus interventions, children are accompanied by adults on a designated route (a core SR2S intervention strategy). The included standalone environmental infrastructure document is relevant to the engineering strategies in SR2S interventions. Similarly, the incentive and gamification documents tap into the encouragement strategies of SR2S interventions. Finally, non-intervention documents were sampled on the basis that these documents provided information on parental perceptions of AST, barriers and facilitators to AST, and children’s mobility more generally. The documents containing information on barriers and facilitators to AST, for example, were used to identify patterns in contextual factors and outcomes, and to strengthen the support for mechanisms identified in the SR2S and non-SR2S intervention documents. A more in-depth overview of the characteristics of included documents has been provided in Supplementary File S3.

### 3.2. CMOCs and Program Theory

In total, 16 refined CMOCs were developed and clustered into the three partial program theories pertaining to the actions of implementor/implementation, children, and parents. Nine mechanisms were contained within these three partial program theories. The implementor/implementation partial program theory comprised the mechanisms of: (1) School Reliance and (2) School Priority. The child partial program theory comprised the mechanisms of: (3) Fun and (4) Pride. Finally, the parent partial program theory comprised the mechanisms of: (5) Perceived Safety, (6) Distrust, (7) Convenience, (8) Perceived Capabilities, and (9) Reassurance. Table 3 summarises the 16 CMOCs in relation to the mechanism and target stakeholder group (i.e., implementors, children, and parents).

The following section provides a brief description of each CMOC contained within each partial program theory and how these partial program theories integrate to build an overarching program theory for how, why, and under what circumstances SR2S interventions worked or not. Direct quotations from primary documents and descriptive data excerpts are presented within quotation marks and square brackets, respectively. As SR2S interventions are multi-component, the results highlight the extent to which it has been possible to link a specific SR2S intervention strategy to a CMOC.

#### 3.2.1. Partial Program Theory One: Implementor/Implementation

School staff or implementors who facilitated delivery of SR2S interventions played a key role in implementation fidelity, that is, the extent to which the multi-component SR2S intervention was delivered as originally planned. The mechanisms of school reliance on a facilitator/champion and school perceived priority relative to support emerged as being particularly influential to implementation fidelity. Each mechanism is highlighted below in relation to the driving contextual factors and outcome of implementation fidelity.

##### Overreliance on Facilitator/Reliance on Champion (CMOC 1)

Internal champions/facilitators play a critical role in the implementation of AST interventions (e.g., SR2S) and the promotion of AST to their school community. When SR2S intervention resources were provided to schools with an uncommitted program champion/facilitator (C), management unsupportive of promoting AST (C), or low readiness to support AST initiatives (C), the responsibility of implementation was given to a facilitator who lacked passion for AST or an intervention and/or belief in the intervention’s ability to change behaviour. When schools developed an overreliance on an uncommitted facilitator to implement and promote an intervention, implementation tended to be done with minimal effort (M), which led to certain strategies (e.g., SR2S maps) not being distributed as intended and/or insufficient promotion of the intervention and its resources to the school community (O). This led to low implementation fidelity (O). There was some evidence to suggest that schools reliant on a passionate and committed internal champion/facilitator could overcome these barriers and drive intervention implementation. Reliance on a champion meant that aspects of the intervention, such as the SR2S maps, would reach children and parents, and positively contribute to implementation fidelity. The importance of a champion was highlighted by a school representative in Buttazzoni, Coen, and Gilliland’s study [37]:

“[Who the] facilitator is, can, in my experience, make or break the success of the school travel plan. I think it is very important that they believe in the program. If it is just kind of a thing that they have to do… I don’t find that it is nearly as successful as someone who really believes in the program, gets it, is passionate about it, and drives it and makes it happen.”

There was a preponderance of evidence in favour of schools being over reliant on an uncommitted facilitator versus reliant on a passionate and committed champion to drive the process. Overall, it appeared that school support for AST interventions, such as SR2S, may have been an afterthought and created problems with adequate implementation.

##### Perceived Priority Relative to Support (CMOC 2)

Whether schools perceived AST to align with their priorities impacted the resources (e.g., staff time) granted to promote AST and AST interventions. When SR2S resources were provided to schools that had a management unsupportive of AST (C), low readiness to support AST initiatives (C), and/or clear competing priorities (C), SR2S interventions were perceived as low priority ‘add-on’ initiatives (M) and, therefore, assigned limited school resources (O), thus compromising implementation fidelity (O). This situation was highlighted by Crawford and Garrard [39]:

[…schools appeared to have other important issues to deal with, which meant that “add-on” programs like the Ride2School program received less attention than other key priority issues… these schools appeared to require greater levels of support and resources to implement the program…]

Due to the lack of priority for SR2S interventions and accompanying managerial support and resources (e.g., time) (C), the staff responsible for implementation became overwhelmed by the demand placed upon them to implement and promote AST interventions effectively while meeting the targets set by senior school management for other higher priority issues (M). The combination of these school level factors contributed to low implementation fidelity (O), as highlighted by Buttazzoni, Coen, and Gilliland [37]:

[Overall, the demands of the facilitator role was [sic] acknowledged to be one of the greatest challenges and potential liabilities for program success.]

Staff may be able to cope with and overcome the demand of implementing SR2S interventions; however, there was little evidence of such adaptation in the included documents.

##### Summary of Partial Program Theory One: Implementor/Implementation

Key mechanisms that emerged within the school implementation partial program theory were overreliance/reliance on a facilitator/champion and perceived priority relative to support. Within particular school contexts, these two mechanisms influenced the primary outcome of SR2S implementation fidelity. The level of implementation fidelity observed contributed to what SR2S intervention strategies were implemented and how well they were promoted to children and parents. The evidence suggests that schools struggled to implement SR2S interventions with fidelity (i.e., one or more components may not have reached children or parents), therefore limiting the intervention impact on children’s engagement in AST.

#### 3.2.2. Partial Program Theory Two: Child

Children engaged in AST to have fun and out of a sense of pride. Fun emerged as a multidimensional mechanism with different contextual drivers. Having fun and feeling a sense of pride contributed to the outcome of children’s motivation to engage in AST.

##### Fun (CMOCs 3–5)

Having fun while actively travelling to and/or from school was an important factor for children’s motivation. Within the articles reviewed, children discussed AST as being fun for different reasons; either through socialising, engaging with nature, and/or showing off.

For children, having additional time to socialise before the school day started enabled them to talk, play games, and make a positive start to the day, which made AST fun (M). It was not clear whether having fun through socialising was linked with a specific SR2S intervention strategy. However, the presence of a friend or sibling to actively travel to school with (C) was highlighted as a critical contextual factor for children’s fun and the promotion of children’s motivation to engage in AST (O). As was discussed by Hinckson [44]:

[The children enjoyed walking to school… it gave them the opportunity to be with their friends, play games, talk about “things” that mattered to them such as “Star Wars and Lego”, and arrange play dates.]

In contexts where children were required to walk to school alone (C), they did not find AST as fun because they were unable to socialise (M), which detracted from their motivation to engage in AST (O). This was highlighted by one child in Hinckson’s study [44], who stated:

“I would rather walk but no one who lives down my street walks. If I walked I would have to do it by myself.”

Children also having the opportunity to engage with nature, through feeding animals (e.g., ducks), picking flowers, and learning about their natural surroundings, made them perceive AST as fun (M) and promoted their motivation to engage in AST (O). As one child from Donnellan, Egli, and Smith’s study stated [56]:

“I like walking past trees because they look nice. They have flowers that I really like and sometimes I want to pick some flowers and take them to school.”

From the literature reviewed, it was not clear if children having fun through engaging with nature was linked with a specific SR2S intervention strategy. Moreover, it was also unclear whether the SR2S maps were designed with consideration of children having opportunities to engage with nature. However, children’s access to open footpaths located away from main roads (C), and routes to school with the presence of nature aspects (C), were important contextual factors. When children were required to actively travel to school using routes near busy roads (C), the noise from vehicles and the lack of nature aspects detracted from their motivation to engage in AST (O).

SR2S interventions that used activity tracking strategies, either standalone or as a component of broader competition or challenge-based strategies, provided children with the opportunity to track their personal AST behaviour (e.g., their total distance travelled) and show it off to their friends. Some children, particularly those who lacked motivation for AST (C) and/or were competitive in nature (C), found it fun to track the number of trips they made, add to their total, and visually display their AST behaviour to show off to friends (M). This promoted children’s motivation to engage in AST (O). As was discussed by Rutberg and Lindqvist [57]:

[Parents reported how their children thought it was fun to put stickers on the board for every kilometer they walked or biked to and from school; they were trying to meet the challenge and were motivated to use AST.]

##### Pride (CMOC 6)

Feeling a sense of pride for AST and/or an intervention was another factor that promoted children’s motivation to engage.

SR2S interventions that used incentive-based intervention strategies, such as giving children an item of clothing to wear (e.g., a cap with the intervention’s logo), provided children with the opportunity to showcase their involvement and to receive acknowledgement for their efforts (M). These types of incentive-based intervention strategies worked particularly well for children that did not participate in many extracurricular activities (C), and/or those that had social and/or academic difficulties (C). Children feeling a sense of pride for AST or a specific intervention promoted their motivation to engage in AST (O). This was highlighted by Rutberg and Lindqvist [57]:

[They expressed that their children were proud of being part of this project… Halfway into the intervention, they received a cap with the text ‘I walk and bike to school’ (in Swedish), which served as an acknowledgement of being part of the project. This made them engage in more walking and biking…]

##### Summary of Partial Program Theory Two: Child

Emergent within the child partial program theory were the mechanisms of fun and pride. These two mechanisms, when activated under particular contextual conditions, were key drivers for SR2S interventions to achieve the primary outcome of children’s motivation to engage in AST. The findings suggest that there is no single pathway to children’s motivation and that different contextual factors influence children’s motivations to engage in AST. For some children, multiple pathways may drive motivation and for others one pathway may predominate. Importantly, whether children’s motivation translated into engagement in AST was determined by their parents and household or family circumstances.

#### 3.2.3. Partial Program Theory Three: Parent

As the primary caregivers for children, parents (or guardians) are the ultimate decision makers for supporting children’s motivation to engage in AST. Several contextual factors embedded in the household, school, and neighbourhood were identified as influencing parent’s perceptions of safety, distrust, convenience, and their children’s capabilities to engage in AST. Parents needing to feel a sense of reassurance that their child had arrived at school safely was an additional mechanism influencing the decision to support their children’s motivation to engage in AST.

##### Perceived Safety (CMOCs 7–10)

How parents perceived the safety of AST influenced whether they allowed their children to engage. Perceived safety emerged as a multi-faceted mechanism. From the literature reviewed, parents discussed perceiving AST as being safe and unsafe for different reasons; either because of the traffic and road environment, stranger danger, bullying, or safety in numbers.

The traffic and road environment presented a major safety concern for parents, particularly in neighbourhoods that had high-speed/high-traffic roads (C), lacked supportive pedestrian infrastructure for AST (C) and/or safe crossing facilities (C). Parents perceived these environments to be unsafe for their children to actively travel because of the danger posed by vehicles and the risk of their children being injured (M). These concerns were particularly prominent among parents of children that had to travel to school independently (i.e., without adult accompaniment) (C). These safety concerns negatively influenced parental decision making (O), which detracted from children’s engagement in AST (O). As was highlighted in Nikitas, Wang, and Knamiller’s study [47]:

[Parents claimed that they have witnessed repeated incidents of cars refusing to stop at pedestrian crossings en route to school and cars regularly driving down the wrong side of the road to get around stationary traffic. Two parents said that concerns over road safety led them to drive to school as they felt their children were not provided with a safe walking environment.]

Despite SR2S interventions providing engineering improvements (e.g., upgraded crossing) and education strategies (e.g., road safety education), it was unclear whether the implementation of one or both of these strategies was sufficient enough to change parental perceptions of safety within these contexts, particularly when these perceptions were as entrenched as suggested in the reviewed studies. However, in contexts where the built environment within neighbourhoods was supportive of AST (C), parents perceived the neighbourhood environment to be safer for their children to traverse (M), positively influencing parental decision making (O), which promoted children’s engagement in AST (O). As was captured by a representative from an AST exemplar school (i.e., high rate of AST) in Hawley et al.’s study [76]:

“… And the infrastructure is there, and we’ve got the bike paths or the footpaths to do it safely.”

Parents’ fear of stranger danger, in contexts where children were required to travel to school alone or in small groups independently (C), using a route that had low community activity (C), or high incidences of crime (C), made them perceive their children to be at greater risk of interacting with strangers and being abducted (M). This fear was compounded, as there were no adults around to see what happened and/or provide help if needed (C). This perceived risk of abduction negatively influenced parental decision making (O), which detracted from children’s engagement in AST (O). As one parent from Donnellan, Egli and Smith’s study stated [56]:

“… I start worrying about [him] because he’s not on a main road if anything happens, no one’s going to see what happened if you know what I mean…”

From the literature reviewed, it was unclear if any of the current SR2S intervention strategies could address parental safety concerns of stranger danger within these contexts.

Peer-to-peer bullying was another safety concern for parents with younger or smaller children travelling to school alone or in small groups independently (C), where older or bigger children (e.g., those attending high school) were present along the route (C). This made parents perceive their children to be at greater risk of being victims of bullying, intimidation, and/or theft (e.g., bicycles and other personal property) (M). This perceived risk led parents to perceive AST as unsafe for their children, which negatively influenced parental decision making (O), detracting from children’s engagement in AST. A parent shared their concern in Ahlport et al.’s study [61]:

“It can also just be scary if the groups of middle school kids come and they’re intimidating to the little kids”

It was not clear if any of the current SR2S intervention strategies could overcome the lack of parental perceived safety because of bullying within these contexts.

Importantly, parents’ perception of AST being safer in numbers was a driver for children’s engagement in AST. Parents perceived AST to be safer for their children if they could travel as part of a group (C), or by using a route popular with other children (C). SR2S interventions that used and promoted school-specific SR2S map strategies increased the number of children using the same route to travel to school. Having other children present, either directly (e.g., travel partners) or indirectly (e.g., using the same route), improved how parents perceived the safety of AST because children could look out for and help each other when needed (M), which positively influenced parental decision making (O), promoting children’s engagement in AST. As was stated by one parent in Faulkner et al.’s study [62]:

[“if it’s a whole bunch of kids walking up at the same time” because there is “safety in numbers”]

Children using routes to school that had high community activity (C) was another critical contextual factor for parental perception of AST being safer in numbers. Routes to school being popular with adult members of the community, either for commuting to work, dog walking, and/or exercise, was identified as important for improving how parents perceived the safety of AST. These adults, although not associated with walking children to school, provided informal supervision to the children using the route and could safeguard against elements that parents find unsafe (e.g., strangers) (M), positively influencing parental decision making (O), which promoted children’s engagement in AST (O). As one parent from Francis et al.’s study stated [63]:

“I always feel more secure if I know there’s other people who might keep an eye on my kids or might help my kids if they need help”

##### Distrust (CMOCs 11–12)

Parental distrust was an important factor for whether children engaged in AST. Distrust emerged as a complex pathway that linked into parental perceived safety. From the literature reviewed, parents discussed distrust in different ways; either because of members of the community or child behaviour.

Parents described an inherent distrust towards unknown members of their community and the perceived safety risk they posed to their children. Families that were new to a neighbourhood (C), lived in a neighbourhood of low socioeconomic status and/or high crime (C), belonged to a minority group (C), or lived in a community that lacked social cohesion (C), had inherently high levels of distrust, as they lacked social connections with residents who could potentially interact with their children en route to school (M), making them perceive it to be unsafe (M). Such high levels of distrust towards members of their community negatively influenced parental decision making (O), detracting from children’s engagement in AST (O). As one parent stated in Donnellan, Egli and Smith’s study [56]:

“… I don’t find it as safe as what it used to be when I was growing up. I remember walking around and it didn’t matter, but now you just can’t trust anyone, really, especially if you don’t know them.”

It was unclear if any of the current SR2S interventions strategies could overcome parental distrust of their community. However, families that lived in communities with greater social cohesion (C) were more trusting of members of the community to be around their children because they knew them (M), fostering safety in numbers (M), which positively influenced parental decision making (O), promoting children’s engagement in AST.

Parents distrust of their children and/or their children’s friends to behave themselves when travelling to school, particularly when travelling to school independently (C), and/or if their children or their friends were prone to misbehaving (C), was highlighted as a major safety concern for parents. This risk of children misbehaving led parents to perceive AST to be unsafe because their child could be distracted at important moments (e.g., when crossing the road), and created a sense of unpredictability as to whether their child could be led astray (e.g., deviating from the route and arriving at school late) (M). It was not clear if any of the current SR2S interventions strategies could overcome parental distrust, thus the risk of children misbehaving negatively influenced parental decision making (O), which detracted from children’s engagement in AST (O). As said by a parent in Nikitas, Wang, and Knamiller’s study [47]:

“… At least when it’s you with them you can control things a little bit more but when they meet up with other kids you just have no idea what’s going to happen.”

In contrast, it is conceivable that for some parents, trusting their children and their children’s friends to behave appropriately on the way to and/or from school was not an issue, thus contributing towards children’s engagement in AST. However, there was limited evidence highlighting this within the documents reviewed, as parents’ discussion of distrust was dominant.

##### Convenience (CMOC 13)

How parents perceived the convenience of AST relative to the demands on their time played an important role in children’s engagement. Parents perceived AST to be less convenient than passive modes of travel (e.g., car) because of the time required in the morning. Parents who worked and would not let their children travel independently (C), and/or had multiple things to do before work (e.g., preparing children that attend different schools) (C), perceived driving their children to school to be the most convenient option because it required less time and organisation, and they could save time by chaining trips together and continuing their journey directly to work (M). The perceived inconvenience of AST negatively influenced parental decision making (O), which detracted from children’s engagement in AST (O). As was summarised by the authors of the Oregon Transportation Research and Education Consortium final report [73]:

[For many parents, the timing of the school start and their work made it impossible to bike or walk with their child, and so they preferred to drop their child off on their way to work…]

It was unclear if any of the current SR2S interventions strategies could overcome the parental perceived inconvenience of AST within these contexts. In contrast, parents who did not experience these multiple demands (C), and/or lived close to their child’s school (C), were more inclined to engage in AST with their children because it was perceived as more convenient than battling the traffic in a car (M), particularly if the infrastructure was favourable (C). As summarised by Nikitas, Wang and Knamiller [47]:

[For some parents walking was the preference because they lived close to the school. Some parents said it is quicker to walk than drive due to road congestion and parking constrains.]

##### Perceived Capabilities (CMOCs 14–15)

Parental perceptions of their children’s capabilities were an important driver for children’s engagement in AST. Some parents discussed perceiving their children to lack the physical, knowledge, and/or skill capabilities required to actively travel in a safe manner.

Parents of young children (e.g., pre-school—grade 3), and/or children with disabilities (C), perceived their children to lack the physical capabilities required to use AST, particularly in contexts where their children would be required to travel a far distance (C), traverse a neighbourhood with hilly topography (C), and/or carry a heavy backpack to school (C). Parents perceived that, because of the physical challenges posed in these contexts, their children risked exposure to physical harm (e.g., muscle strain) or being too tired to focus after they arrived at school (M). It was unclear if any of the current SR2S interventions strategies can overcome how parents perceived their child’s physical capabilities. This lack of perceived capability negatively influenced parental decision making (O), which detracted from children’s engagement in AST (O). As stated by a parent in Nikitas, Wang, and Knamiller’s study stated [47]:

“School is about 30 minutes’ walk. He’s four and he’s a tiny four so it’s quite a long walk really. When my son gets older he might be able to walk further.”

Parents also had to consider whether their child had the skill and knowledge capabilities to actively travel safely. When children were required to travel to school independently (C), in a neighbourhood with high-speed/heavy-traffic roads (C), and poor infrastructure and crossing facilities (C), parents perceived their children to lack the skill and knowledge capabilities to navigate and to make safe decisions, and instead had concerns about their children getting lost on the way to school or crossing the road at an unsafe location and getting injured (M). This negatively influenced parental decision making (O), which detracted from children’s engagement in AST (O). As one parent from Ahern et al.’s study stated [60]:

“I don’t think she can cross this main road at the bottom on her own… I don’t think she’s really got that concept yet of crossing the road properly…”

Despite SR2S interventions providing education strategies (e.g., road safety education), it was not clear whether these strategies alone could change how parents perceived their child’s skill and knowledge capabilities in these contexts. However, when SR2S interventions used encouragement strategies (e.g., walk to school day events) in conjunction with education strategies, in contexts where parents had time flexibility to accompany their children in the morning (C), these event days provided parents with the opportunity to try AST without commitment to continued engagement. In doing so, children could demonstrate what they had learnt, and parents realised that their children had the capability to actively travel to school safely (M), positively influencing parental decision making (O), which promoted children’s engagement in AST (O). As one parent within Rutberg and Lindqvist’s study stated [57]:

“I was skeptical [sic] before, and felt like the study would force us to change our way of managing transport to school, but afterwards, when I realized that she managed to go to school by herself with a friend, then it was only positive…”

##### Reassurance (CMOC 16)

The feeling of reassurance influenced parental decision making as to whether they let their children engage in AST. For parents who were unable to accompany their children to school because of work commitments (C), letting their children travel independently (C), particularly within a neighbourhood that they perceived to be unsafe (C), created a lack of reassurance that their children would arrive to school safely using AST (M). This is often the case in schools that do not routinely or consistently contact parents if their child does not arrive at school (C). This lack of reassurance made parents feel uneasy and interfered with their daily activities, which negatively influenced parental decision making (O), detracting from children’s engagement in AST (O). As was discussed by one parent in Forsberg et al.’s study [53]:

“If I wouldn’t be able to know if they had arrived safely to school, then I wouldn’t be able to relax at work, I need some kind of confirmation to be able to unwind, you know, ok now they are at school, now I can breathe out”

From the literature reviewed, it was not clear if any of the current SR2S intervention strategies provided parents with the reassurance needed to let their children engage in AST within these contexts.

##### Summary of Partial Program Theory Three: Parent

Parents’ decision making related to their children’s engagement in AST is a complex process. The findings suggest that there may be multiple considerations that influence their decision. For many parents, it may not be a singular mechanism, but a combination of perceived safety, distrust, convenience, child’s capabilities, and reassurance that conspire together to influence their decision within household circumstances and aspects of the social and built environment. Whilst children may be motivated to engage in AST for fun and pride, parents’ concerns act to ‘trump’ children’s motivations. SR2S intervention strategies do not comprehensively address many of the perceived barriers to AST, which are overwhelmingly featured in the reviewed literature.

#### 3.2.4. Overarching Program Theory

**Figure 3:**
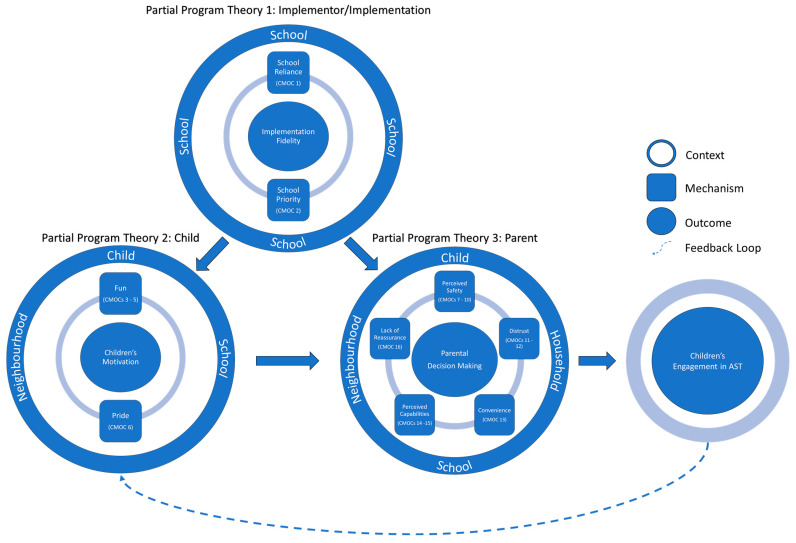
Overarching Program Theory.

Presented in Figure 3 is a visual representation of the refined overarching program theory that explains how, why, and under what circumstances SR2S interventions influenced school children’s engagement in AST in high-income countries. The refined overarching program theory consolidates the complex relationships between the 16 refined CMOCs and the three partial program theories (i.e., implementor/implementation, child, and parent).

The overarching program theory suggests that the extent to which SR2S intervention strategies are implemented as originally intended (i.e., fidelity) provides the context for whether children and parents become aware of and engage with the SR2S strategies provided. Although intervention success hinged on program champions and schools sufficiently resourcing and supporting implementation, the evidence suggested that AST interventions are often an afterthought and resulted in schools’ reliance on an uncommitted facilitator. Even if a committed facilitator/champion is present at a school, the lack of prioritisation for AST relative to other school priorities leaves the facilitators/champions feeling overwhelmed. There was lesser evidence showcasing a supportive school context for implementing the multicomponent strategies of a SR2S intervention. The review leaves an impression that schools experience a number of barriers when it comes to implementing a multicomponent intervention such as SR2S, which raises important questions on whether (and which) components have been implemented with fidelity.

When implemented and promoted successfully, SR2S intervention strategies were able to motivate children to engage in AST by making it fun (although this was mostly contextually driven) and instilling them with a sense of pride. Children’s motivation for AST, however, could be ‘trumped’ during the parental decision-making process, which was heavily influenced by perceptions of safety, distrust, convenience, their child’s capabilities, and the need to be reassured that their child arrived at school safely. The major drivers of parental decision making for children’s engagement in AST were mostly contextual and implicate factors emanating from the child, household, school, and/or the social and built neighbourhood environment. If parental decision making was positively influenced, and children’s engagement in AST permitted, this reinforced children’s motivation to engage. Despite the current SR2S strategies being able to motivate children, they appear to not address, or lack sufficient potency to address, many of the parental contextual barriers to children’s engagement in AST. For example, the implementation of a singular major infrastructure change proximal to a school (e.g., a school crossing) may change the parental perceived safety for children using that route (or multiple if routes funnel together), but is unlikely to change the parental perceived safety for children that use different routes, or of the neighbourhood as a whole.

The overarching program theory presented here provides a detailed picture of the complexity linking SR2S interventions with the outcome of children’s engagement in AST. The process is not static, but instead dynamic, with child and parent mechanisms changing with continual changes in context. For example, the parent mechanism of perceived capabilities may change as the child ages and transitions through school because they are perceived to have greater physical capabilities as they age.

## 4. Discussion

SR2S interventions have been implemented in Australia and other high-income countries to promote children’s engagement in AST. This review found that SR2S interventions can promote children’s motivation to engage in AST, but there are important preconditions. School commitment to implementation can support children’s motivation, but the extent to which children’s motivation translates to engagement in AST is influenced by the gatekeeping role of parents. Parental perceptions of safety are the most prominent mechanism influencing children’s engagement in AST. While broadly supporting the existing literature, this review’s application of realist logic allows for clarification of key contextual factors in relation to mechanisms of children’s engagement in AST. Previous reviews have focused only on intervention effectiveness and have not explained how, why, or under what contextual conditions SR2S interventions have worked or not. Instead, there has been a tendency to informally allude to the mechanisms of change (e.g., stranger danger). In using a theory-driven methodology, we have drawn on theoretically relevant documents from the body of published peer-reviewed and grey literature for SR2S interventions that otherwise would have been excluded from systematic reviews based on quality appraisal (e.g., studies using non-experimental designs). In doing so, we have been able to build, test, and refine an overarching program theory that depicts the different pathways to children’s engagement in AST. This work addresses Pang et al.’s [19] call for more theory-informed research to inform the development, execution, and evaluation of AST interventions.

This review adds new findings to the existing AST literature by unpicking and formalising the complexity of the gatekeeping role that parents play in children’s engagement in AST [82]. As highlighted in our overarching program theory, SR2S interventions can motivate children to want to engage in AST for the aspects of fun and pride. However, whether their motivation results in actual engagement is contingent on parental decision making. Herein lies the gatekeeping role. The review findings suggest that parental decision making appears to be dependent on several child, parent, family/household, school, and neighbourhood factors that drive parental perceptions of safety, distrust, convenience, their children’s capability, and the need for reassurance of their child’s safe arrival. If parents perceive a specific route, or AST more generally, to be unsafe, inconvenient, or not physically possible for their children to use, or they are not provided with the necessary reassurance from their child’s school, then it is unlikely that they will enable their child’s engagement in AST, irrespective of their child’s motivation. SR2S interventions need to consider the diverse factors that shape parental decision making during the planning phase. This may require the broadening of the current suite of SR2S intervention strategies offered (e.g., walking school bus).

Perhaps unsurprisingly, and consistent with previous research [11,12], parental perceived safety was found to be critical for children’s engagement in AST. SR2S interventions are distinguishable from other AST interventions by the school specific SR2S maps that promote designated ‘safe’ routes that avoid busy roads and intersection crossings. This review challenges the underlying assumption of SR2S interventions that simply steering children away from busy roads and crossings makes routes ‘safe’. This study is novel for highlighting the underlying complexity of parental perceptions of safety, with the traffic and road environment forming only one part of the multidimensional mechanism. Importantly, parents can perceive a given route to be more or less unsafe than other routes for a variety of reasons (e.g., peer-to-peer bullying), and their judgement is dependent on several route- and neighbourhood-specific social and built environmental factors, some of which are quite intractable (e.g., high neighbourhood crime, older children present along the route, or lack of neighbourhood cohesion). For example, a SR2S map may promote a route that is located away from a busy road, which addresses the parental perceived safety concern from the traffic and road environment. Nonetheless, if that route is also popular with older children, parents may perceive it to be unsafe due to the potential inadvertent exposure to bullying. Or, if one of the promoted routes uses a footpath with low community activity, then parents may have greater concerns of stranger danger, especially if their child is travelling alone. Moreover, it is probable that within highly specific contexts, SR2S interventions may address all parental perceived safety concerns. However, for many parents, the lack of context-specific tailoring means that SR2S interventions do not, or are not potent enough to, address all parental perceived safety concerns, which challenges the underlying assumption that all promoted routes are equally ‘safe’. The notion that parental perceived safety is multidimensional, and that many of the contextual drivers for parental perceived safety are route and neighbourhood specific, must be acknowledged in the design of future SR2S interventions. This extends to considerations for neighbourhood socioeconomic status, which was featured only briefly in the analysis in relation to CMOC 14 Parental Distrust (Table 3).

To date, evaluations of SR2S interventions have primarily focused on children’s AST behaviour, with less attention given to intervention implementation. A small number of studies have assessed implementation either standalone [37,38] or within larger behaviour change evaluations [40,41,43]. These evaluations, however, provide limited information for site-level implementation. Using the limited evidence, this review suggests that school support and readiness to implement the education and encouragement components of SR2S interventions are low. Consequently, the assigned school staff or program champion responsible for implementation can become easily overwhelmed due to the lack of resources and demand of other competing priorities and/or interventions. This apparent struggle that schools experience with implementing the multiple components of SR2S interventions appears indicative of partial or complete implementation failure [83] and may, in part, contribute towards the inconsistent AST outcomes reported in the reviewed interventions. That said, without greater systematic assessment of site-level implementation, interpreting SR2S intervention outcomes is problematic. Therefore, greater site-level assessment of implementation for SR2S interventions is needed.

Implemented independently of the school by local council/government transport agencies are the SR2S engineering strategies. These strategies, which are recognised as the main component of SR2S interventions, see the installation of major infrastructure changes to the built environment, such as a new children’s road crossing or bicycle infrastructure, and minor changes, such as the installation of signage. From the small number of studies that reported on engineering implementation [40,41,43], we, unfortunately, could not easily link specific infrastructure changes at the route or school site level, as reporting was often aggregated across multiple sites. However, implied from the information reported, and given the substantial cost associated with major infrastructure projects [84] and inclusive enrolment policies resulting in a large number of schools enrolled, it appears that only a single major infrastructure change is often implemented per school, supported by multiple minor infrastructure changes [43]. As SR2S interventions promote multiple ‘safe’ routes per school, it is probable that only a singular route may benefit from any change made. Consequently, this may address parental safety concerns for the traffic and road environment of that route, but will likely have little impact on addressing these concerns for the other promoted routes, or of the neighbourhood as a whole. Moving forward, SR2S practitioners and policymakers may want to assess eligibility of schools’ neighbourhoods for major infrastructure changes on a “needs” basis. This would allow for the redirection of funding as part of a more targeted approach to addressing parental traffic and road safety concerns for school neighbourhoods most at need, by enabling multiple major infrastructure changes to be made per school, providing coverage across all promoted routes.

Overall, the overarching program theory raises questions about whether SR2S interventions can promote and sustain children’s engagement in AST, hence the inconsistency in intervention outcomes reported. This review is novel for highlighting that several mechanisms and associated contextual drivers appear to be unaddressed by the current suite of SR2S intervention strategies. Included in these are parental convenience, the bullying of younger children by older children due to shared paths, topography (e.g., hilliness), community distrust, and schools failing to reassure parents of their children’s arrival. SR2S interventions may benefit from integration within a comprehensive school health strategy that manages bullying or provides younger students with proactive coping strategies when confronted by older children, or by embedding SR2S interventions within a Healthy Community Initiative with an AST focus to overcome parental distrust among the community. Supporting the Community Preventive Services Task Force’s considerations for implementation of AST interventions [85], Table 4 provides context-specific strategy recommendations that can be used to inform future SR2S interventions, and AST interventions more broadly. Furthermore, intervention implementors should consider undertaking a needs assessment to tailor intervention strategies to the characteristics of the school population, families’ situational circumstances, and the school neighbourhood environment. Concept mapping, a grassroots participatory program evaluation planning tool, could be used with school staff and families to rate the importance of local AST concerns and potential strategies to address these concerns [86]. This methodology would allow for these concerns to be examined, for example, by role (families vs teachers), parental SES, and/or children’s age and gender. With this information, implementors could tailor the intervention strategies to engage children and families more effectively.

This review has several strengths. To ensure the review’s robustness, we systematically applied the methodological guidance outlined in the RAMESES quality standards for realist synthesis. To our knowledge, this review is the first to apply a realist approach to the available published peer reviewed and grey literature for SR2S interventions. Our review had active involvement from a range of stakeholders, including academe, industry, and end users. This allowed for diverse experiences and perspectives on SR2S and AST to be shared, and our findings to be sense checked against their real-world knowledge and experiences.

This review was not without limitations. Intervention reporting within the included documents was poor overall, which made identifying the specific strategy or combination of strategies associated with the 6 E’s framework and related outcomes challenging. In the few studies that assessed implementation, the specific major and minor engineering strategies were indicated in the intervention summaries. However, the individual education and encouragement strategies tended to be lumped under their broad 6 E’s framework heading, which made it difficult to determine exactly what strategies were implemented. A strength of realist methodology is that it uses both quantitative and qualitative evidence in the synthesis process. In the case of this review, the included quantitative evidence for intervention effectiveness provided limited theoretically relevant data, due to eight of the nine included studies having aggregated AST outcomes across multiple sites, which masked site-specific contextual variation. As such, our synthesis has relied heavily on the included qualitative evidence to unpick the contextual factors and mechanisms that influenced children’s engagement in AST. The qualitative data often focused on the parental perceived barriers to children’s engagement in AST, which had a predominantly negative influence on their decision making. This focus is reflected in the review’s findings (Table 3). Additionally, we note that the findings mainly reflect the role of parents in children’s engagement in AST. This is largely due to the underrepresentation of children’s views in the included documents. Given SR2S interventions’ narrow view of safety (i.e., focused on traffic and road safety), it appears that the intervention strategies do not comprehensively address parental concerns and, relatedly, that some barriers are difficult to change. To more adequately account for the full spectrum of variation in children’s engagement in AST, more mixed-method evaluation research is required to identify the facilitators of children’s engagement within evaluated interventions. As this review followed RRR methodology, the literature searching process was not exhaustive. Despite forming an expert advisory panel to assist with the bibliographic database searching and grey literature identification, we acknowledge that documents that could have contributed to the development, testing, and/or refinement of the CMOCs and/or the overarching program theory may have been missed. For example, in the included documents, there was a lack of definitive evidence to generate a CMOC involving child gender. This is likely an important aspect of context.

## 5. Conclusions

By using realist methodology, this review has been able to provide a more nuanced understanding of the factors influencing children’s engagement in AST by identifying the important contextual factors that drive influential mechanisms of change. In doing so, the findings provide practitioners and policy makers with an overarching program theory that can be used to inform the planning, implementation, and evaluation efforts of future SR2S interventions, and adapted to inform other AST interventions.

## Figures and Tables

**Table 1:** Example Search Strategy for Database Search One.

Database Name: Scopus			
Date of Search: 26 March 2020			
Field Codes: Title, Abstract, and Keywords (TITLE-ABS-KEY)			
Search #	Concept	Search Terms	No. of Results
#1	Active Travel	Walking OR Cycling OR Bicycling OR “Active Transport*” OR “Active School Transport*” OR “Active School Travel” OR “Active* Travel*” OR Commut* OR “Independent* Mobil*” OR “Independent* Travel*” OR “Independent Licence*”	515,402
#2	Intervention	“Safe Routes to School” OR “Safe Routes” OR “SRTS” OR “SR2S”	2091
#3	Population	Child* OR “School Child*” OR “Elementary School*” OR “Primary School*” OR Boys OR Girls OR Youth*	3,576,701
#4		#1 AND #2 AND #3	121

**Table 2:** Characteristics of Documents Included in this RRR (*n* = 45).

Characteristics	*n*	%
**Country**		
United States	23	51.1
Canada	6	13.3
Sweden	5	11.1
Australia	4	8.9
United Kingdom	4	8.9
New Zealand	3	6.7
**Document Type**		
Peer reviewed research articles	38	84.4
Grey literature reports	6	13.3
Commentary	1	2.2
**Research Methodology**		
Qualitative	25	55.6
Mixed method	10	22.2
Quantitative	9	20.0
N/A	1	2.2
**Intervention type**		
SR2S only	18	40.0
Walking school bus	4	8.9
SR2S + other	2	4.4
Gamification	2	4.4
Incentives	1	2.2
School travel plan	1	2.2
Standalone environmental infrastructure	1	2.2
Non-intervention	16	35.6

**Table 3:** CMOC Summary.

Partial Program Theory 1: Implementor/Implementation		Supporting References
School Reliance		
CMOC 1—Overreliance on Facilitator/Reliance on Internal Champion	Schools that had an uncommitted internal program champion/facilitator (C), management unsupportive of promoting AST (C), and/or low readiness to support AST initiatives (C), became complacent because they developed an overreliance on the uncommitted program champion/facilitator for intervention implementation (M), which led to low implementation fidelity (O).	[37,38,39,40,41,42,43]
School Priority		
CMOC 2—Perceived Priority Relative to Support	Schools that had a management unsupportive of AST (C), low readiness to support AST initiatives (C), and/or competing priorities (C), perceived AST interventions as ‘add on’ initiatives and assigned resources to higher priority initiatives (M), and the staff responsible for implementation of low priority interventions became overwhelmed because they were not given the time or support to promote AST as well as meeting the demand from other priorities (M), which led to low implementation fidelity (O).	[37,38,39,40,41,42,43]
Partial Program Theory 1: Child		Supporting References
Child Fun		
CMOC 3—Socialising	Children that had friends or an older sibling to walk to school with (C), perceived walking to school as being fun because they could socialise on the way (M), which promoted children’s motivation to engage in AST (O).	[40,43,44,45,46,47,48,49,50,51,52,53,54,55,56]
CMOC 4—Engaging with Nature	Children that had access to open footpaths located away from main roads (C), and/or the presence of nature aspects on the route to school (C), perceived walking to school as being fun because they could engage with nature (M), which promoted children’s motivation to engage in AST (O).	[47,53,56]
CMOC 5—Showing Off	Children that lacked motivation to actively travel (C), and/or were competitive in nature (C), perceived walking to school as fun because they could track and show off their travel behaviour to friends (M), which promoted children’s motivation to engage in AST (O).	[45,57,58,59]
Child Pride		
CMOC 6—Pride	Children with low participation in extracurricular activities (C), and/or social or academic difficulties/issues at school (C), felt a sense of pride for their involvement and having their efforts acknowledged (M), which promoted children’s motivation to engage in AST (O).	[45,57,58,59]
Partial Program Theory 3: Parent		Supporting References
Perceived Safety		
CMOC 7—Traffic Safety	In neighbourhoods that had high-speed and heavy traffic roads (C), that lacked supporting infrastructure and/or maintenance (C), parents perceived AST to be unsafe for their children because of the risk that they might get injured (M), negatively influencing parental decision making (O), which detracted from children’s engagement in AST (O).	[43,46,47,48,50,51,52,53,54,56,57,60,61,62,63,64,65,66,67,68,69,70,71,72,73,74,75,76]
CMOC 8—Stranger Danger	When children had access to open footpaths located away from main roads (C), that had low community activity (C), or lived in a neighbourhood with high levels of crime (C), parents perceived AST to be unsafe for their children because of the risk of abduction (M), negatively influencing parental decision making (O), which detracted from children’s engagement in AST (O).	[46,48,51,52,53,56,57,60,62,63,65,66,73,74,77,78,79]
CMOC 9—Bullying	Children that walked to school alone or in small groups (C), with the presence of older/high school children along the route (C), made parents perceive AST to be unsafe because of the risk of their child being bullied by the older children (M), negatively influencing parental decision making (O), which detracted from children’s engagement in AST (O).	[51,56,61,64,66,70,73,74,76]
CMOC 10—Safety in Numbers	When children had friends or older siblings to walk to school with (C), on a route that had high community activity (C), parents perceived AST to be safe for their children because friends, siblings, and/or members of the community provided informal supervision (M), positively influencing parental decision making (O), which promoted children’s engagement in AST (O).	[45,51,52,53,61,62,63,65,73,77,78]
Distrust		
CMOC 11—Community/Adults	Parents that were new to a neighbourhood (C), lived in a neighbourhood of low socioeconomic status and/or high crime (C), belonged to a minority group (C), and/or lived in a community that lacked social cohesion (C), were less inclined to trust members of their community to be around their children (M), negatively influencing parental decision making (O), which detracted from children’s engagement in AST (O).	[46,51,56,63,64,65,66,74,75,78]
CMOC 12—Child Behaviour	When children had friends or older siblings to walk to school with (C), and these children were prone to misbehaving (C), parents were less inclined to trust their child/child’s friend to be well behaved (M), negatively influencing parental decision making (O), which detracted from children’s engagement in AST (O).	[46,47,51,53,66]
Convenience		
CMOC 13—Trip Chaining	Working parents that had limited time flexibility in the morning (C), and multiple children that attend different schools (C), perceived passive travel to be more convenient because they could trip chain and go straight to work (M), negatively influencing parental decision making (O), which detracted from children’s engagement in AST (O).	[46,47,48,50,52,53,56,57,60,62,64,65,70,71,73,75,77,79,80,81]
Perceived Capabilities		
CMOC 14—Physical	For parents of young children (C), that had to walk through a neighbourhood with hilly topography (C), lived a far distance from school (C), and/or were required to carry a heavy backpack (C), perceived their children to not have the physical capabilities to walk to school (M), negatively influencing parental decision making (O), which detracted from children’s engagement in AST (O).	[40,43,47,49,61,64,67,79,81]
CMOC 15—Road & Traffic	In neighbourhoods that had high-speed and heavy traffic roads (C), and/or lacked supporting infrastructure/maintenance (C), parents perceived their children to not have the skill or knowledge capabilities to actively travel safely (M), negatively influencing parental decision making (O), which detracted from children’s engagement in AST (O).	[46,47,51,56,57,60,61,62,65]
Reassurance		
CMOC 16—Reassurance	Working parents that had limited time flexibility in the morning (C), perceived their neighbourhood to be unsafe (C), and/or their child’s school did not actively alert parents if their child failed to arrive (C), lacked the reassurance to let their child actively travel because they would not know if they have arrived at school safely (M), negatively influencing parental decision making (O), which detracted from children’s engagement in AST (O).	[46,49,53,57,61,65,77]

**Table 4:** Recommended context-specific intervention strategies by CMOC.

Corresponding CMOC	Intervention Strategy Recommendations
CMOC 3	Providing resources to schools to support the development of a ‘buddy system’ for children to find active travel partner/s Integration of a walking school bus to encourage social interaction and the establishment of friendships with children of different ages
CMOC 4	Providing route-specific information sheets containing information about the natural environment Development and promotion of walk to school games that encourage engagement with the natural environment
CMOC 7	A walking school bus to provide adult supervision when crossing intersections Positioning of crossing guards at high-risk traffic crossings across a neighbourhood to assist with road crossing
CMOC 8	A walking school bus to provide adult supervision on routes that lack community activity Promotion of the SR2S maps to the wider community to encourage greater route activity during school commute times
CMOC 9	A walking school bus to provide adult supervision and safeguard children against bullying Route guardians that patrol high risk segments of routes to safeguard children against bullying
CMOC 11	Community days/events to build social cohesion and connectedness within neighbourhood communities
CMOC 13	A walking school bus to make AST a more convenient option for parents in the morning
CMOC 16	A walking school bus to provide adult supervision and parental reassurance Automated smart technology capable of providing notifications to parents of their child’s arrival at school to provide parental reassurance [87].

## Data Availability

Not applicable.

## Supplementary Materials

The following supporting information can be downloaded at: https://www.mdpi.com/article/10.3390/ijerph19169976/s1, Supplementary File S1: Initial Program Theories; Supplementary File S2: Search Strategy; Supplementary File S3: Document Characteristics.
