# Supplementary material for: Changing Primary School Children’s Engagement in Active School Travel Using Safe Routes to School Interventions: A Rapid Realist Review
Source: Int J Environ Res Public Health. 2022 Aug 12;19(16):9976. doi: 10.3390/ijerph19169976 (PMC9408451; doi:10.3390/ijerph19169976)
Supplement: Supplementary file 1 [file ijerph-19-09976-s001.zip › Supplementary_File_S1.pdf]

Preliminary CMOC 1 – 'It's Safer in Numbers'

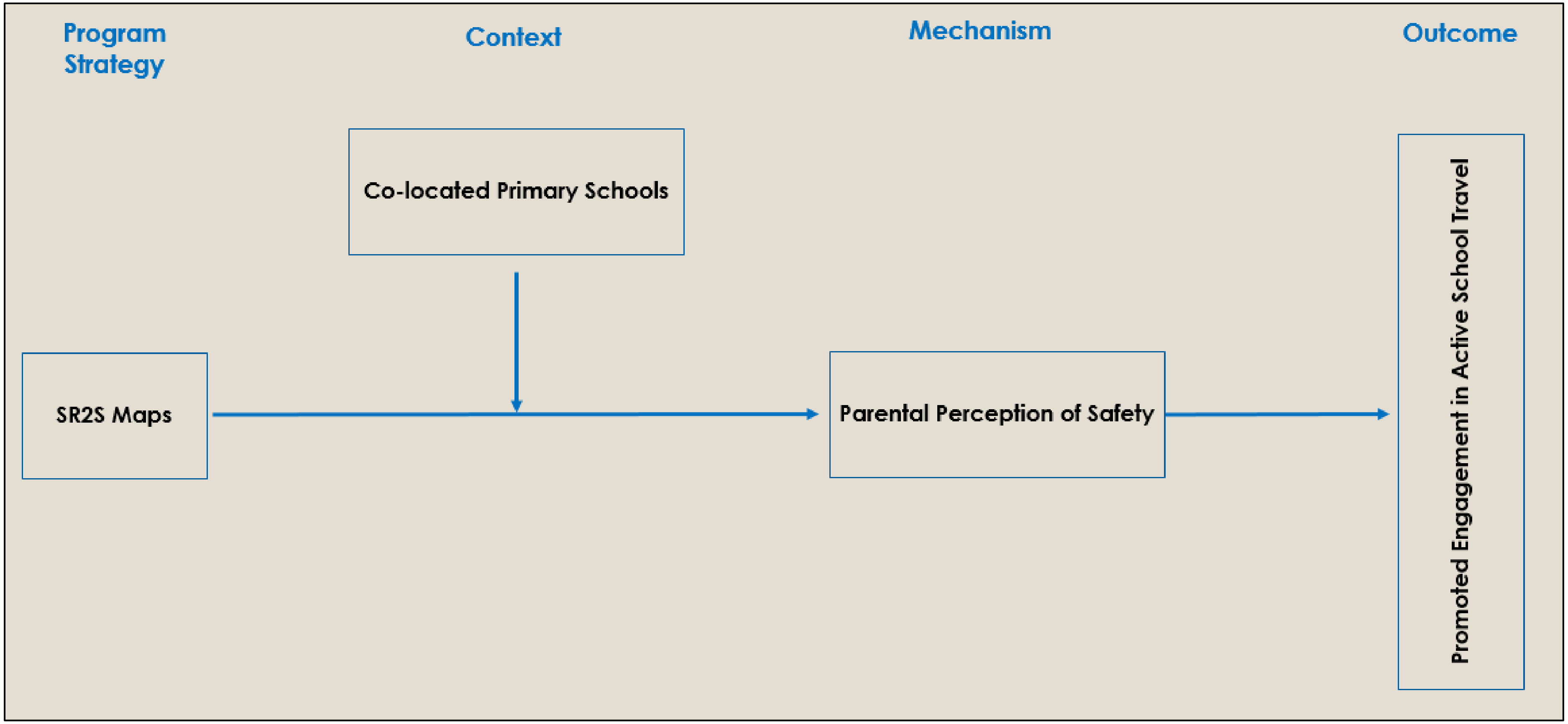

When the safe routes to school maps (Strategy) are introduced into two co-located primary schools (Context), the greater number of active travellers using the same routes to get to school will change parental perception of safety (Mechanism), leading to promoted engagement in AST (Outcome).

Preliminary CMOC 2 – 'It's Safer in Numbers'

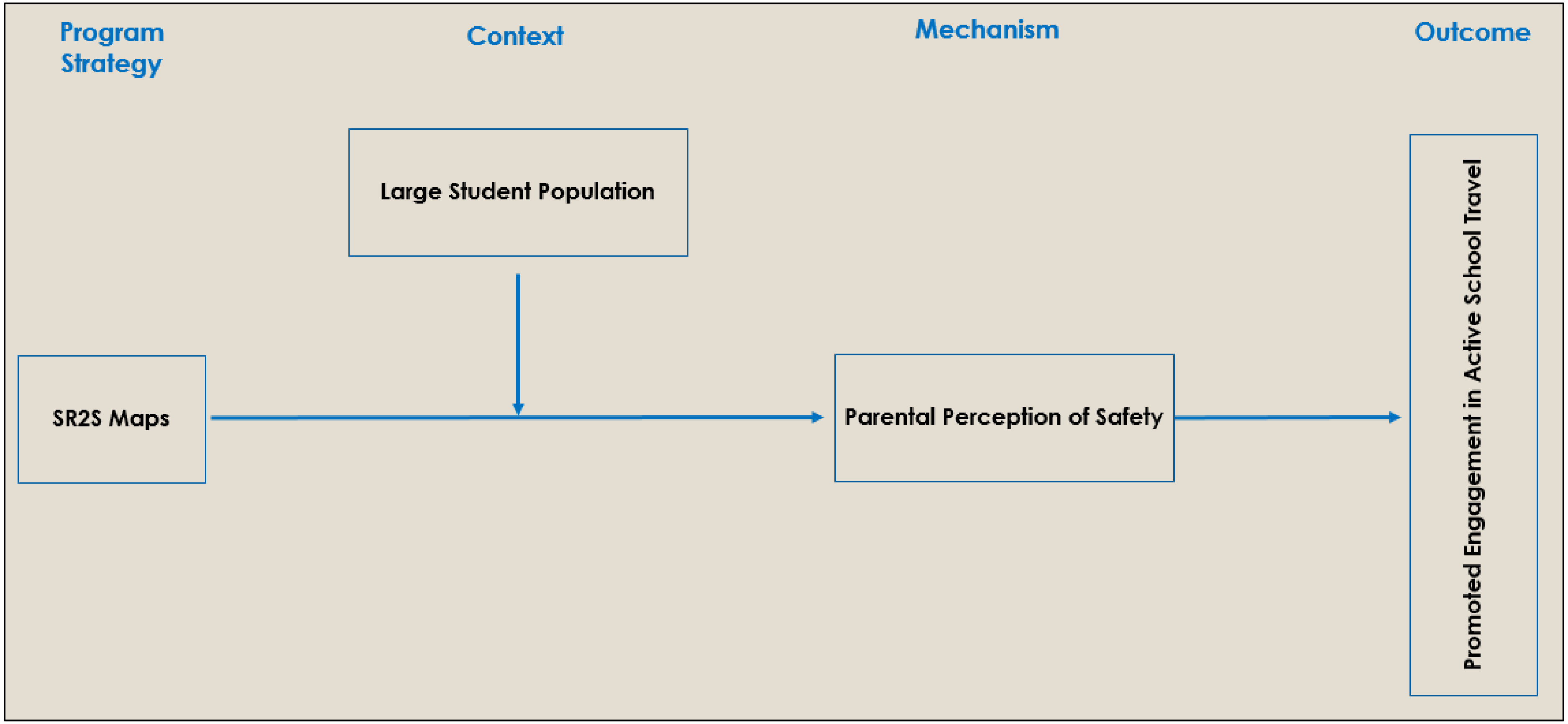

When the safe routes to school maps (Strategy) are introduced into a primary school with a large student population (Context), the greater number of active travellers using the same routes to get to school will change parental perception of safety (Mechanism), leading to promoted engagement in AST (Outcome).

Preliminary CMOC 3 – 'Stranger Danger'

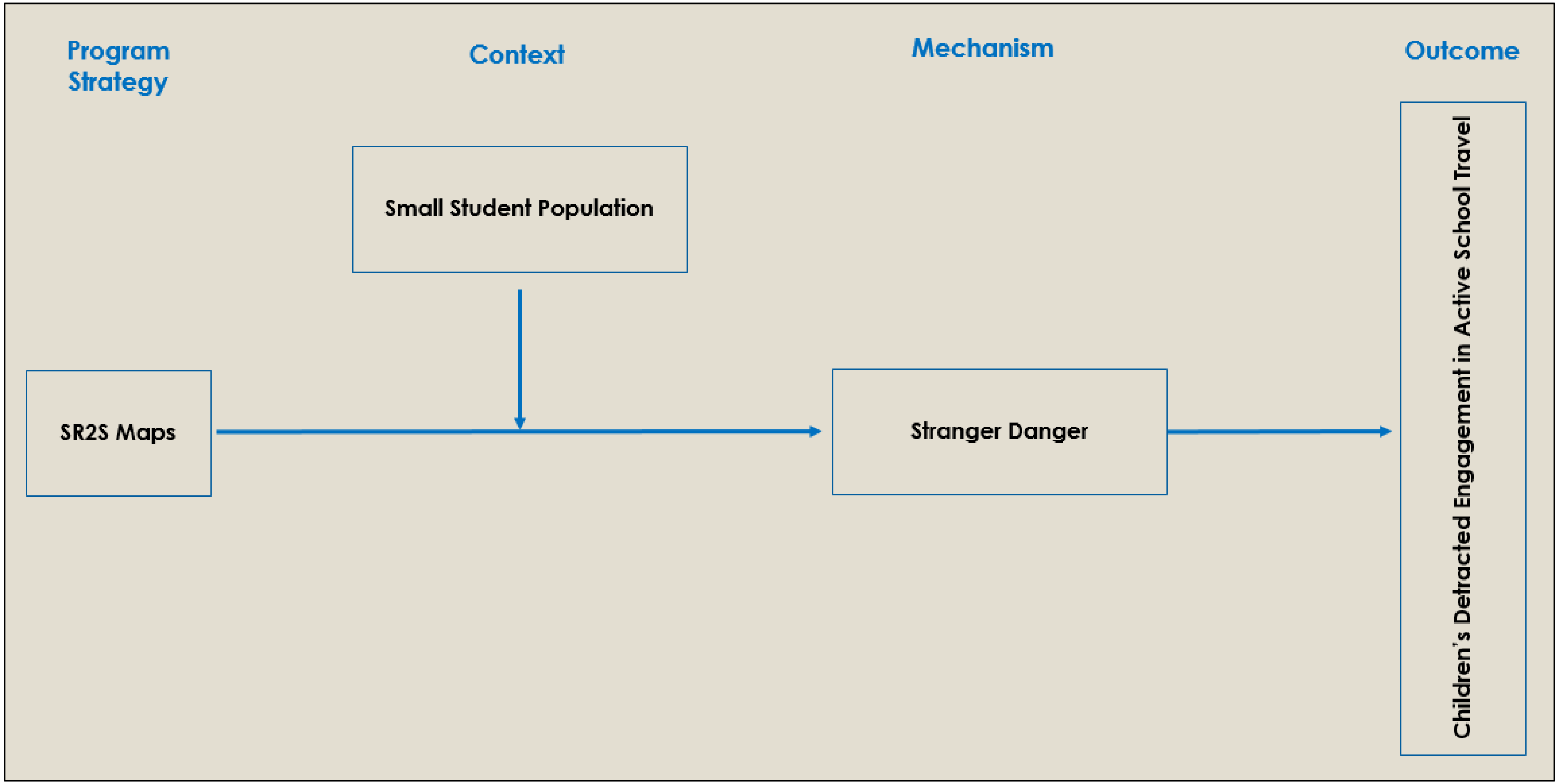

When the safe routes to school maps (Strategy) are introduced into a primary school which is not co-located or has a small student population (Context), there will be too few active travellers using the same routes to get to school, increasing parental concerns about stranger danger (Mechanism), leading to children's detracted engagement in AST (Outcome).

Preliminary CMOC 4 – 'It's Safer in Numbers'

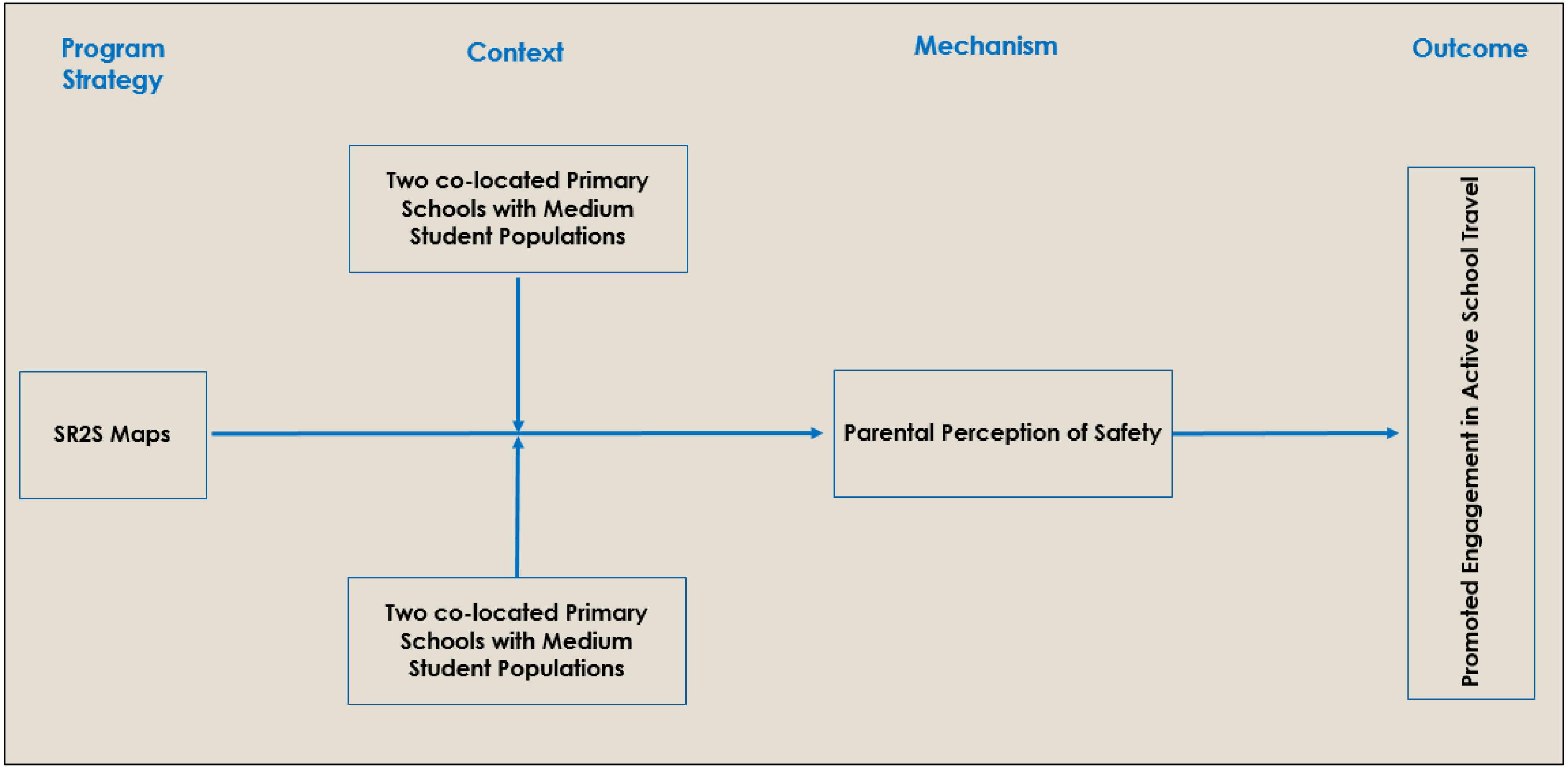

When the safe routes to school maps (Strategy) are introduced into two primary schools with medium student populations that are co-located with a secondary school (Context), or a large primary school co-located with a secondary school (Context), the greater number of active travellers using the same routes to get to school will change parental perception of safety (Mechanism), leading to promoted engagement in AST (Outcome).

Preliminary CMOC 5 – 'Beware of the Older Kids'

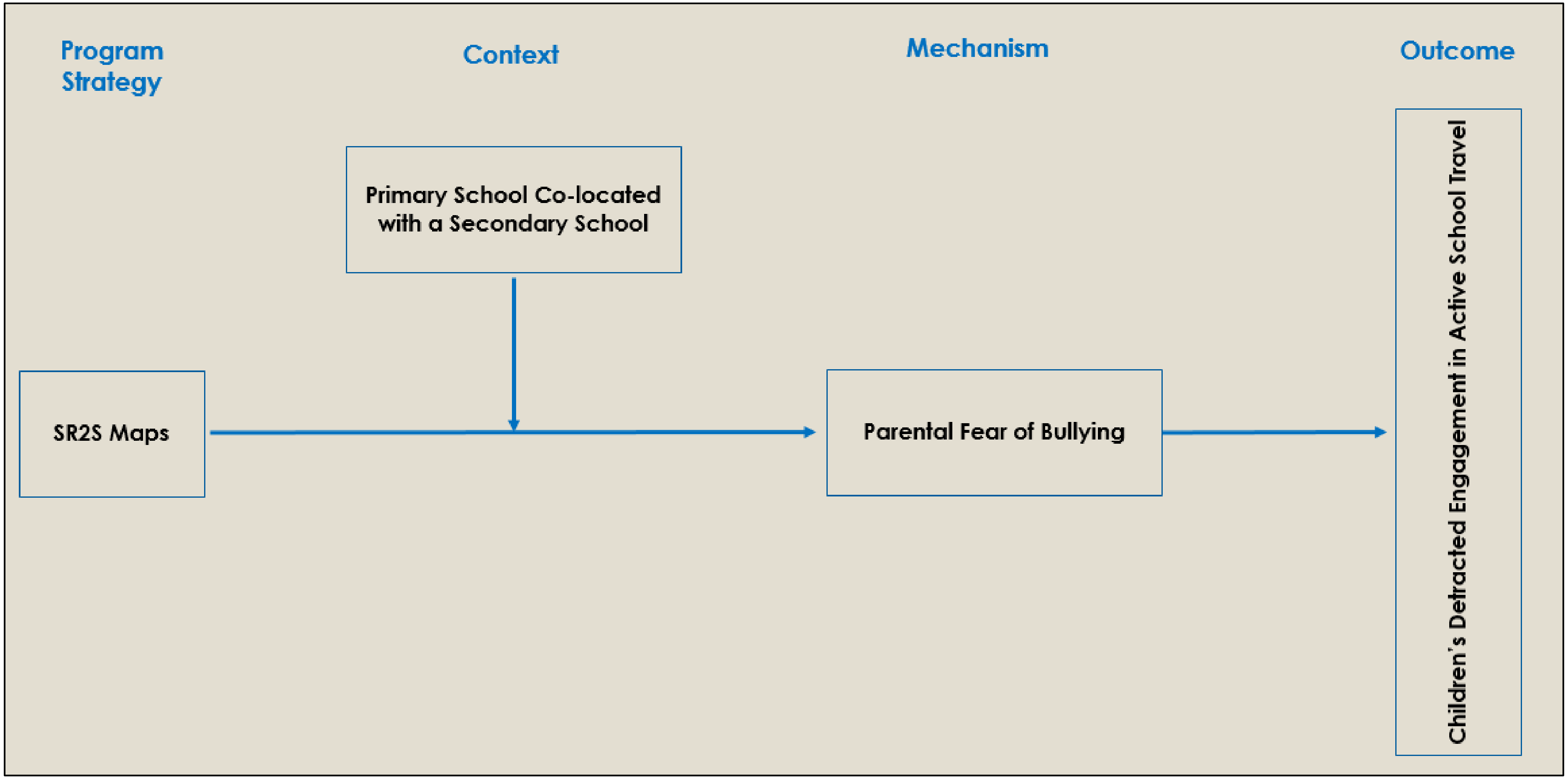

When the safe routes to school maps (Strategy) are introduced into a primary school with a small student population that is co-located with a secondary school (Context), there will be too few active travellers using the same routes to get to school, increasing parental concerns their children may be bullied or peer pressured by older children (Mechanism), leading to children's detracted engagement in AST (Outcome).

Preliminary CMOC 6 – 'It's Already Safe to Walk'

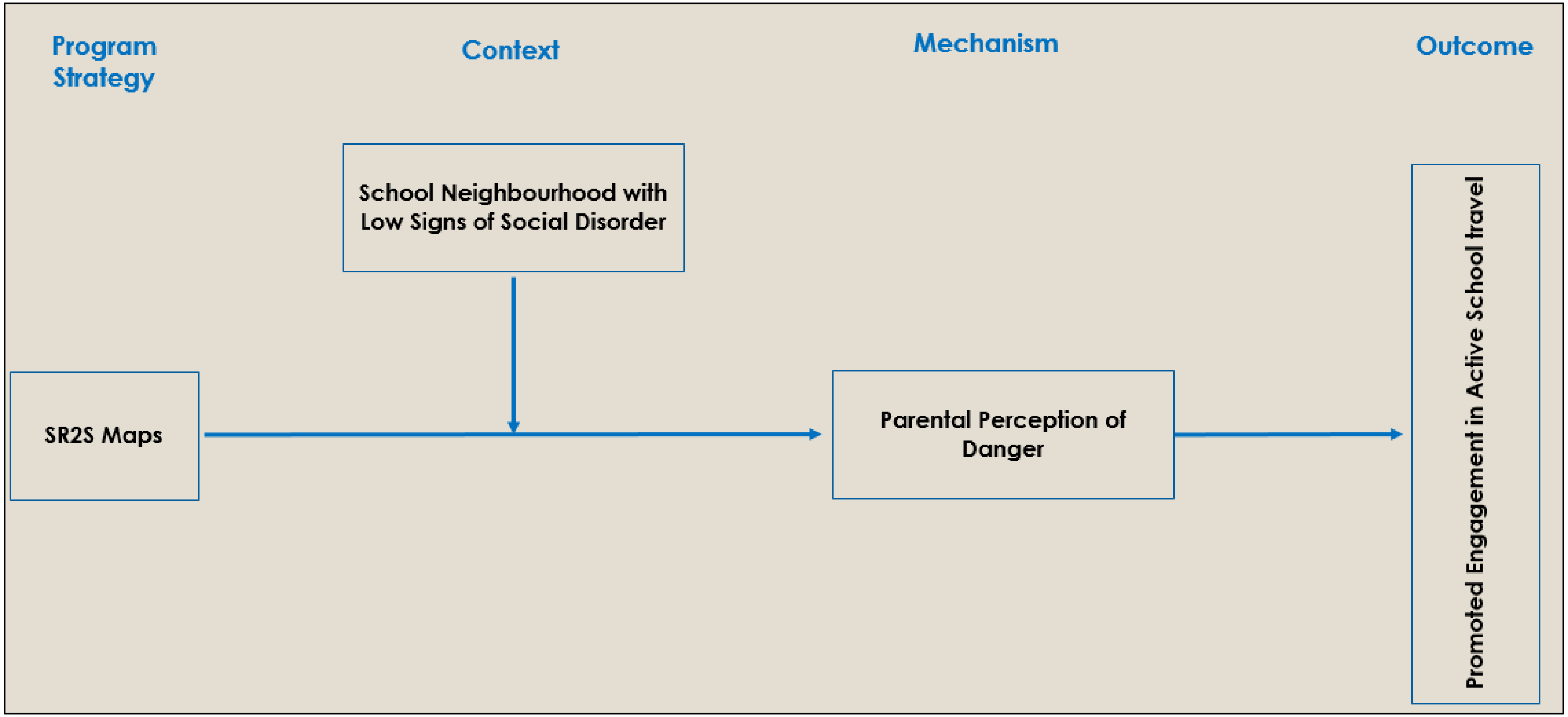

When the safe routes to school maps (Strategy) are introduced into a primary school which neighbourhood has low signs of social disorder (Context), parental perception of the danger of active travel is already low because parents perceive the routes to travel through a safe environment (Mechanism), leading to promoted engagement in active school travel (Outcome).

Preliminary CMOC 7 – 'Eyes all Around'

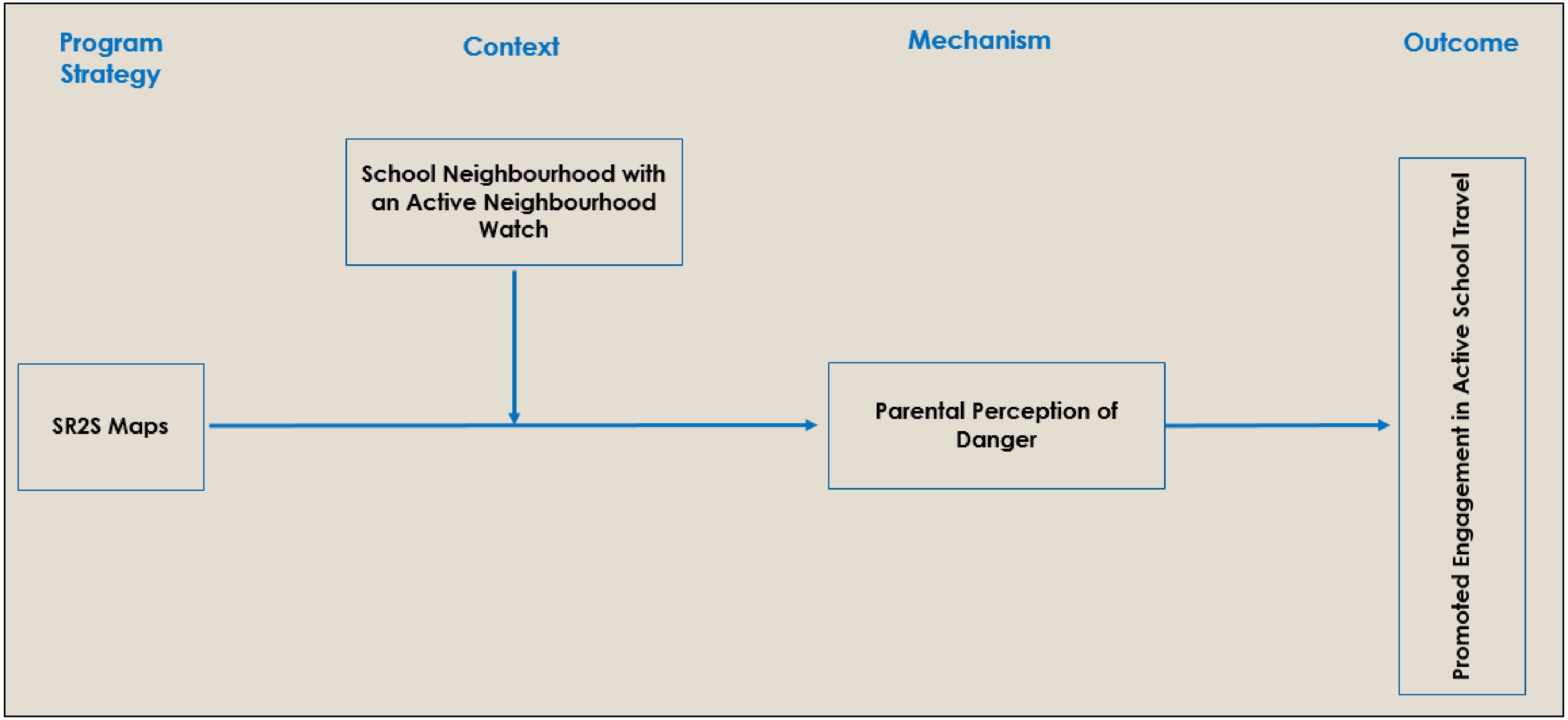

When the safe routes to school maps (Strategy) are introduced into a primary school within a neighbourhood with an active neighbourhood watch (Context), the promoted routes will have passive surveillance from neighbourhood watch members, therefore decreasing parental perception of danger (Mechanism), leading to promoted engagement in AST (Outcome).

Preliminary CMOC 8 – 'Those Roads are too Dangerous'

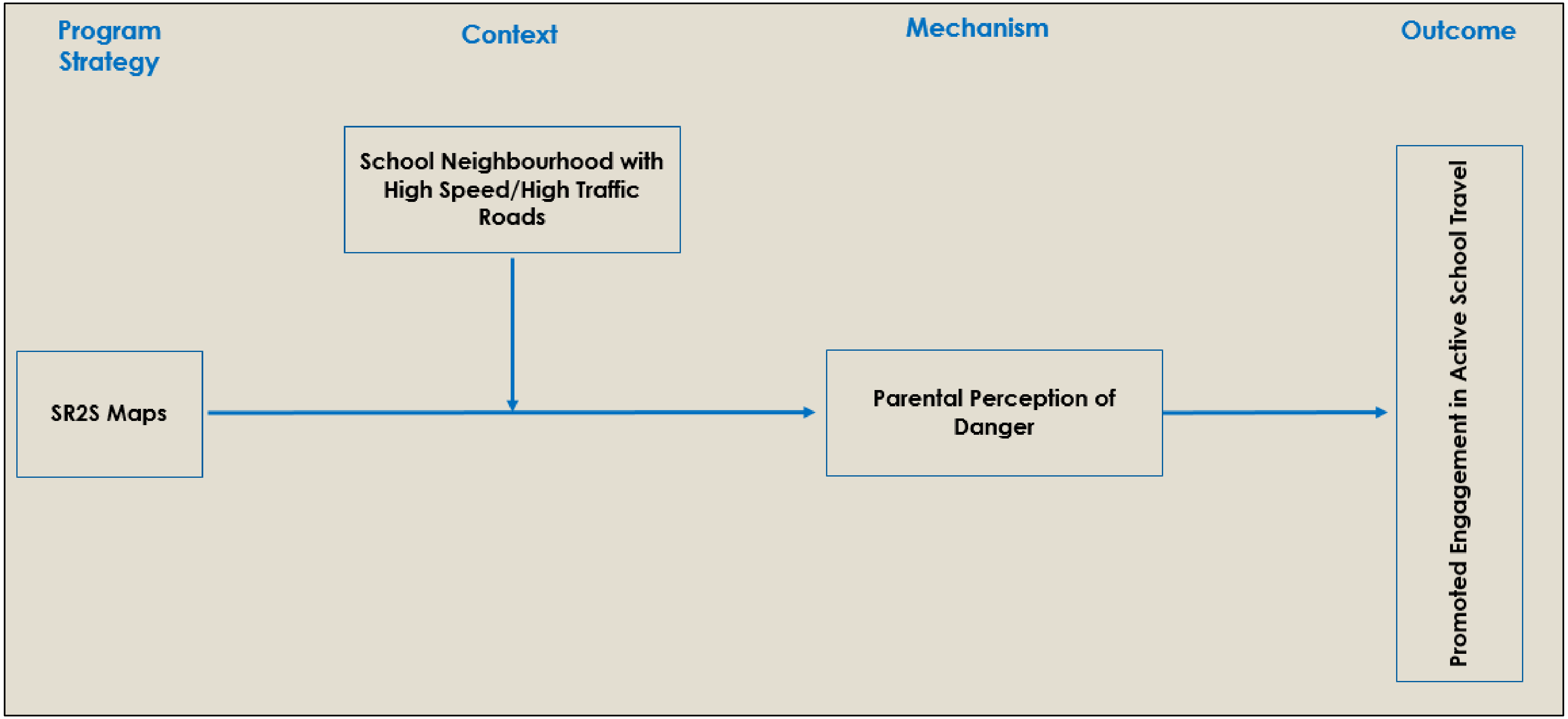

When the safe routes to school maps (Strategy) are introduced into a primary school within a neighbourhood that has a high number of high speed/high traffic roads (Context), children can use the promoted safe routes to avoid the high speed/high traffic roads, therefore decreasing parental perception of danger (Mechanism), leading to promoted engagement in AST (Outcome).

Preliminary CMOC 9 – 'This Neighbourhood's Unsafe'

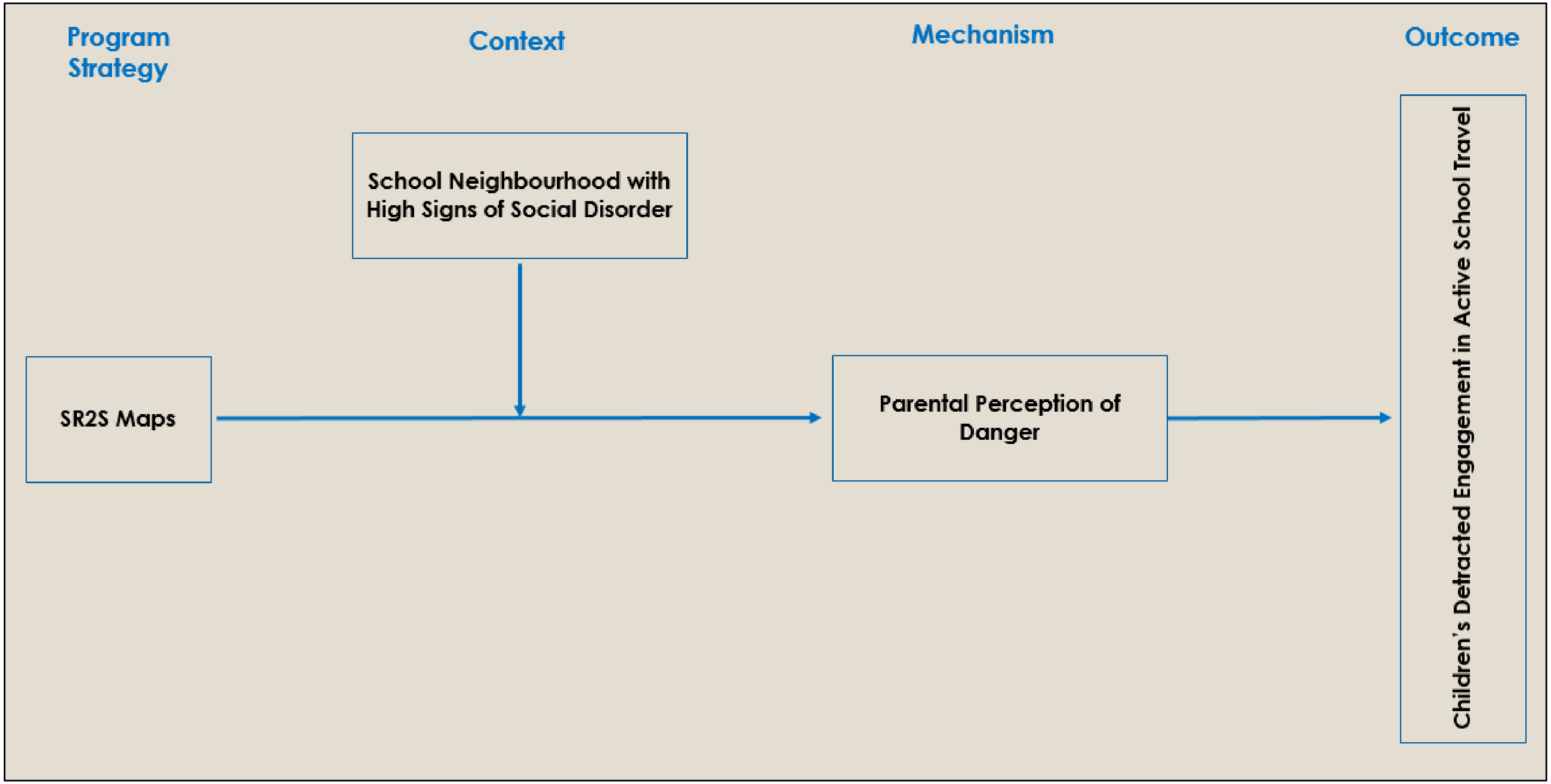

When the safe routes to school maps (Strategy) are introduced into a primary school within a neighbourhood that has high signs of social disorder (e.g., graffiti/litter) (Context), parents will already perceive the routes to school to be in a unsafe environment, therefore not changing parental perception of danger (Mechanism), leading to children's detracted engagement in AST (Outcome).

Preliminary CMOC 10 – 'I'm not Sitting in Bumper to Bumper Traffic'

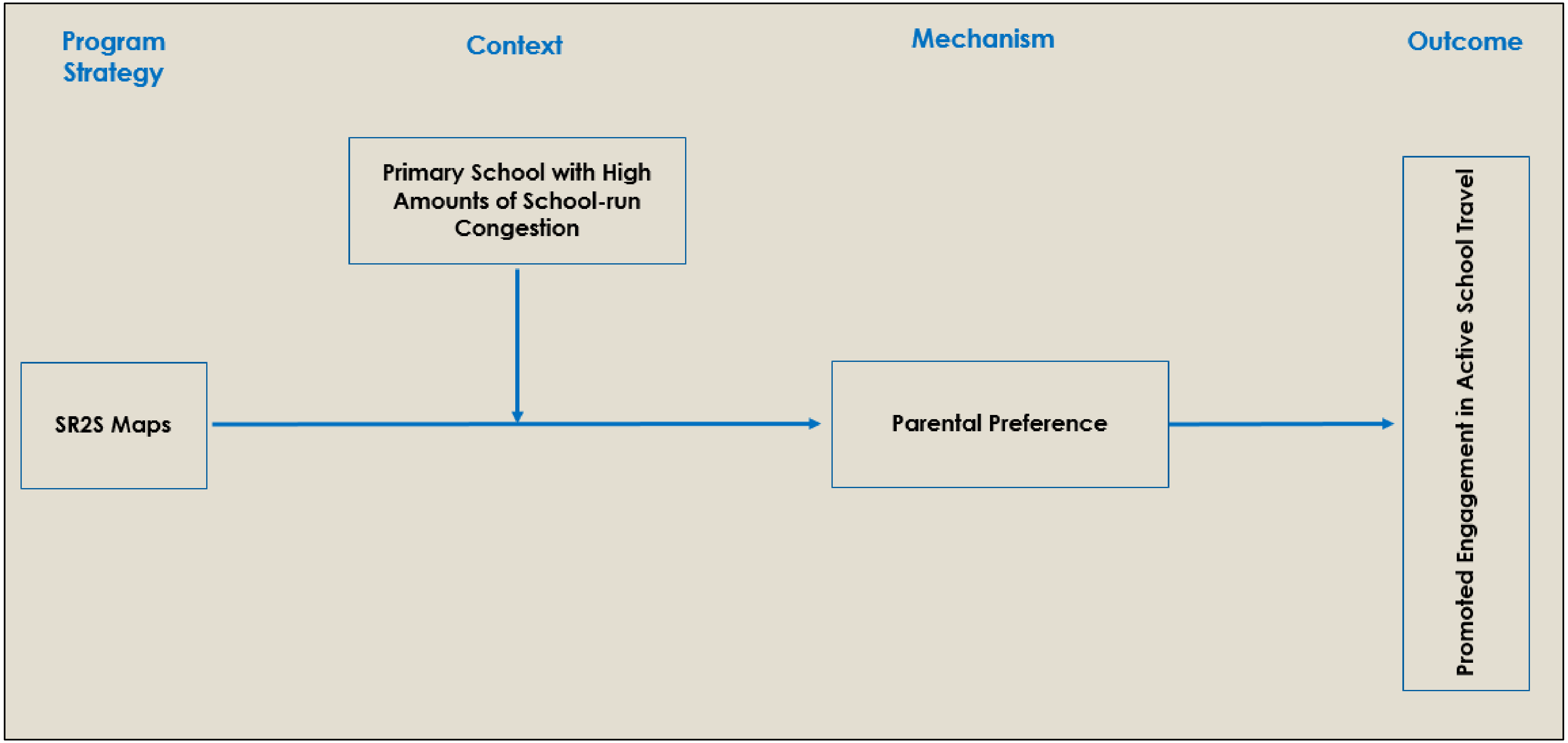

When the safe routes to school maps (Strategy) are introduced into a primary school which has high amounts of school-run congestion (Context), children can use the safe route to school as a safe alternative to passive travel that allows parents to avoid the school-run congestion, therefore, changing parental preference for active travel (Mechanism), leading to promoted engagement in AST (Outcome).

Preliminary CMOC 11 – 'That's too Heavy to Carry'

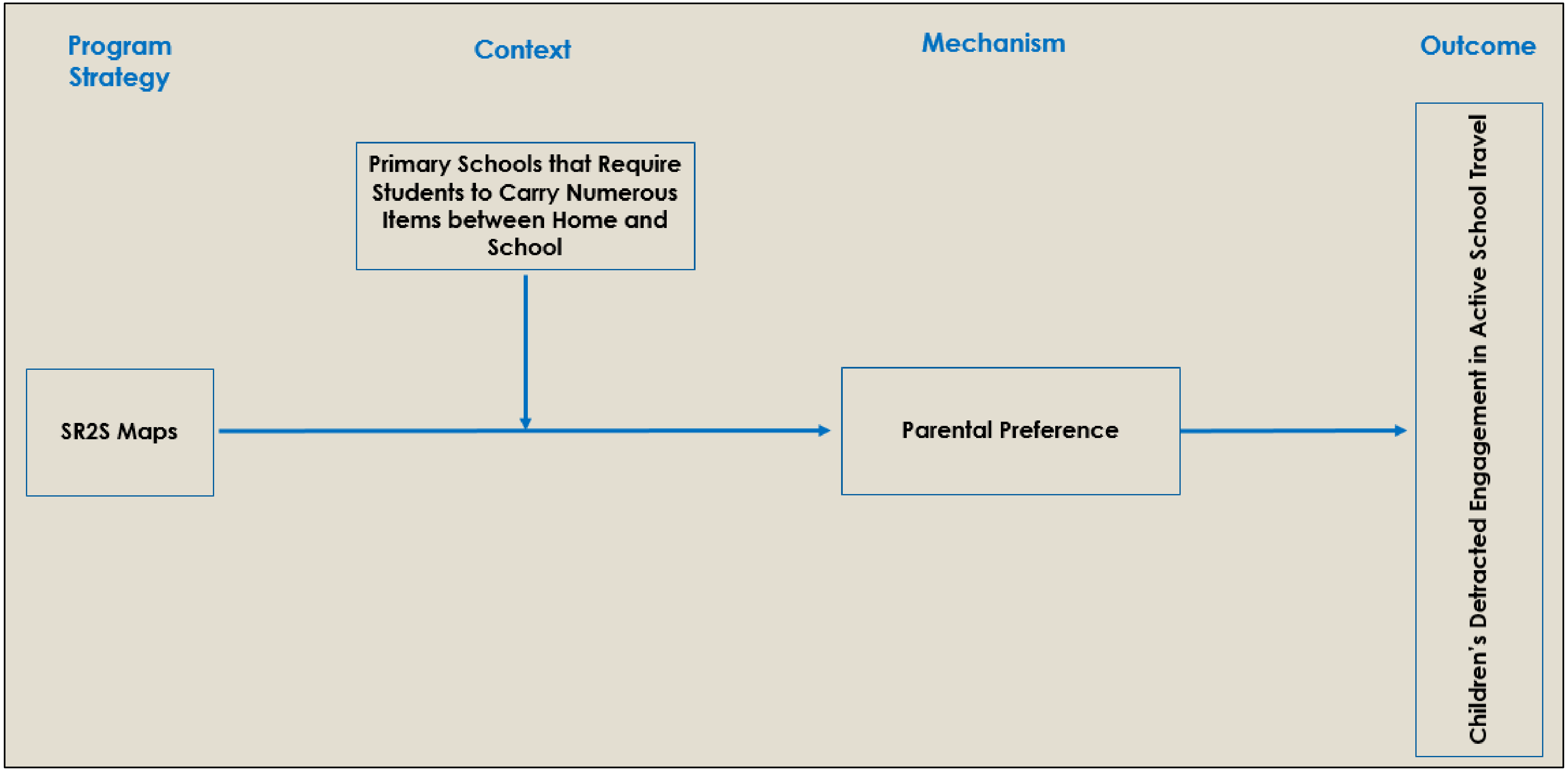

When the safe routes to school maps (Strategy) are introduced into a primary school which requires students to carry numerous items between home and school (e.g., textbooks/exercise books/sports kit) (Context), parents will be concerned for the health of their child(ren) carrying a heavy backpack to school, therefore not changing the parental preference for active travel (Mechanism), leading to children's detracted engagement in AST (Outcome).

Preliminary CMOC 12 – 'It's a Government Program'

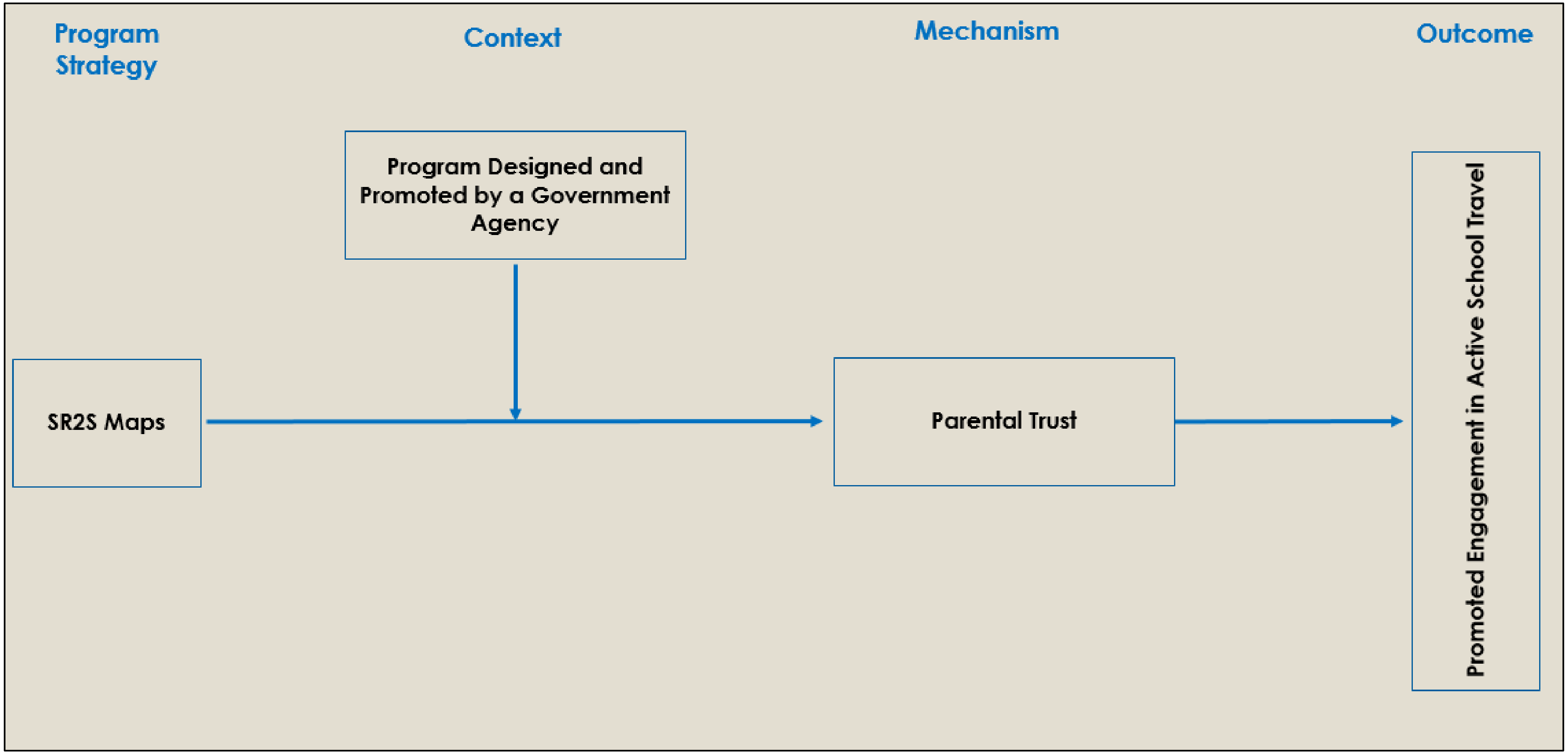

When the safe routes to school maps (Strategy) are designed and promoted into a primary school by a Government agency (Context), parents will recognise that the program is being run by a trusted source, therefore increasing parental trust in the program (Mechanism), leading to promoted engagement in AST (Outcome).

Preliminary CMOC 13 – 'It's not the Only Program you Know'

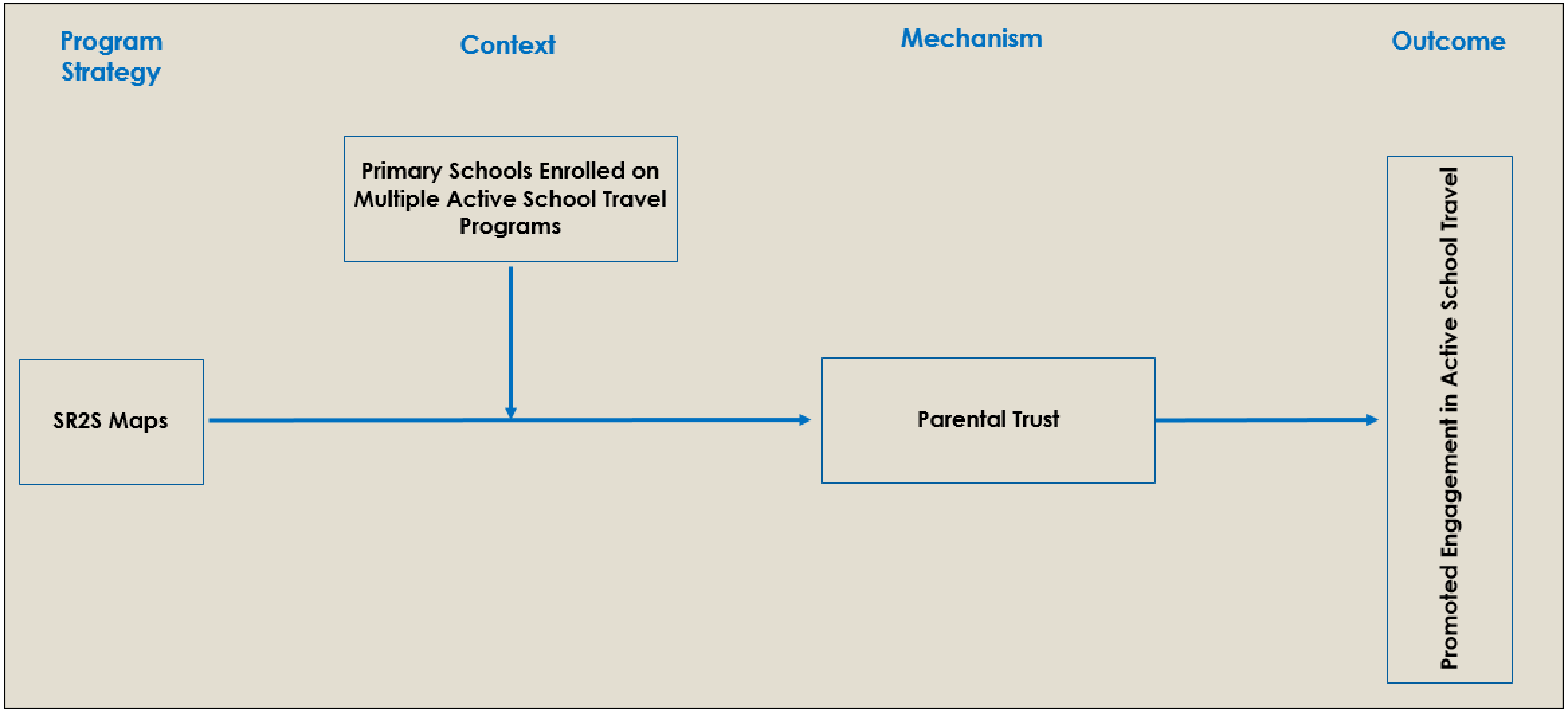

When the safe routes to school maps (Strategy) are introduced into a primary school that is already enrolled on multiple active travel programs (Context), the new resources complement the existing active travel resources available through the other programs, therefore increasing parental trust in the safety of active travel (Mechanism), leading to promoted engagement in AST (Outcome).

Preliminary CMOC 14 – 'It's More Fun with Friends'

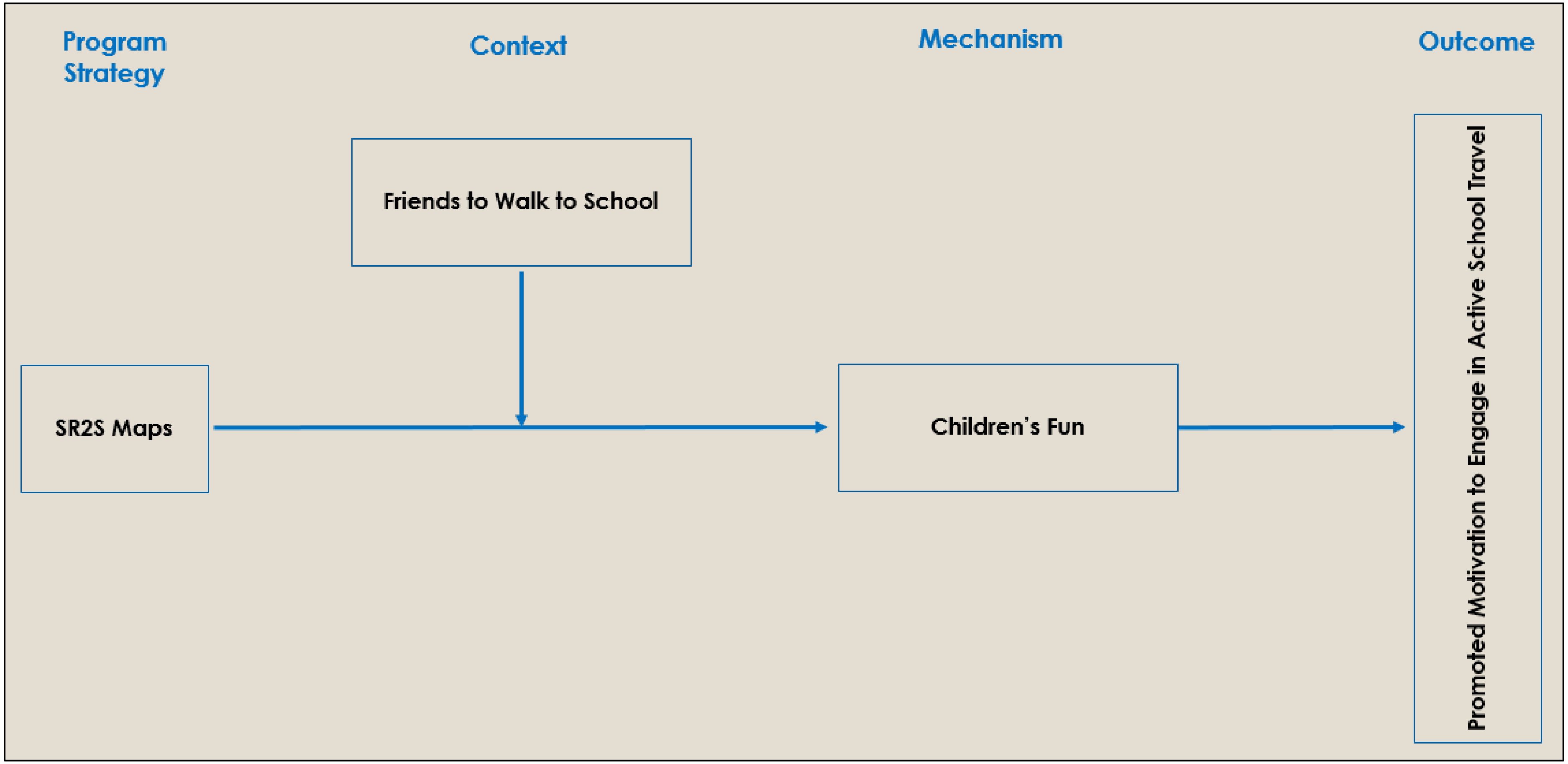

When the safe routes to school maps (Strategy), are provided to children that have friends living close by (Context), the maps encourage children to use the same route to school creating more opportunity to socialise with friends (Mechanism), leading to promoted motivation to engage in active school travel (Outcome).

Preliminary CMOC 15 – 'Look at that Cool Cockatoo'

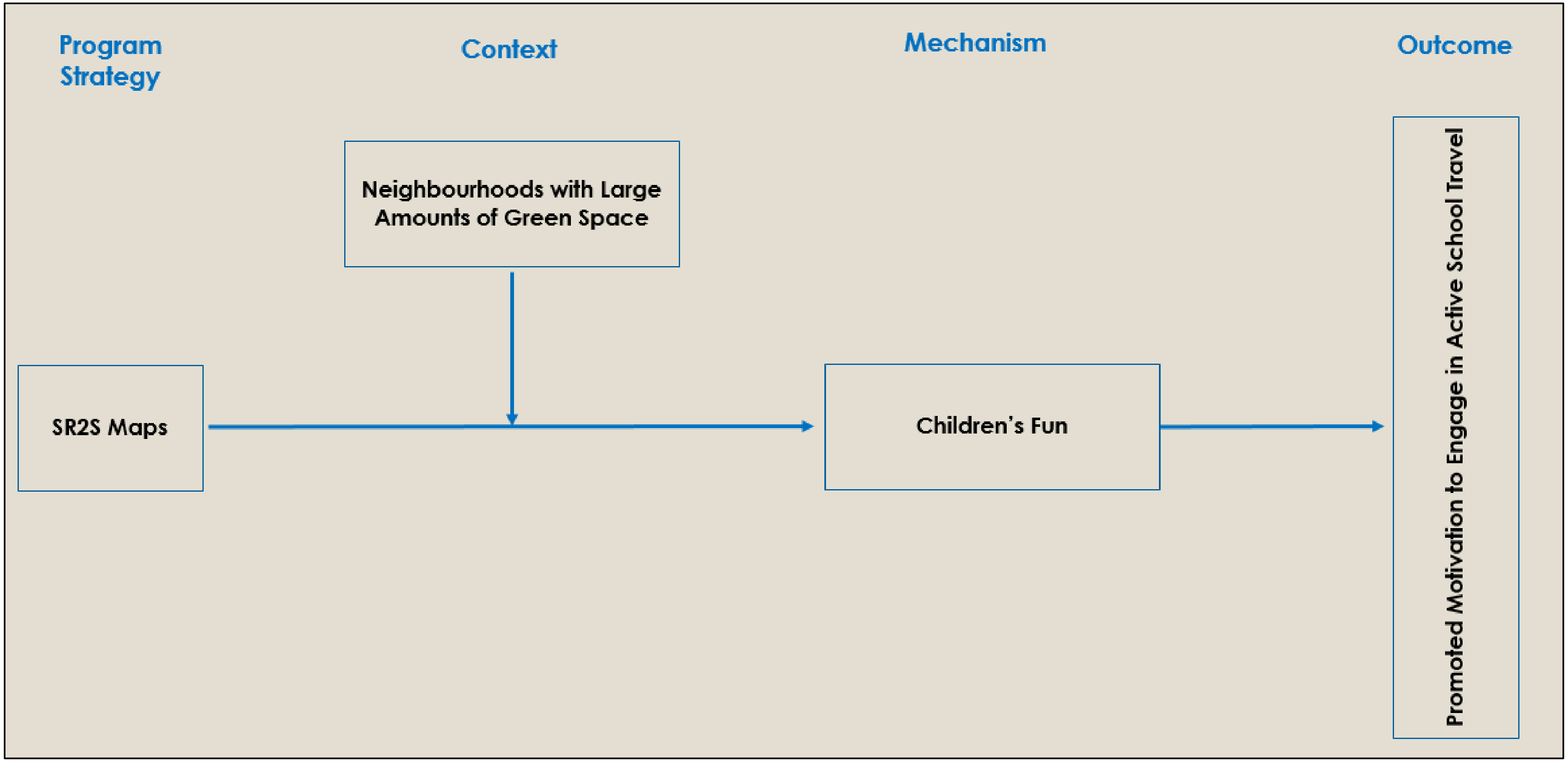

When the safe routes to school maps (Strategy), are provided to children that live in a neighbourhood with large amounts of greenspace (Context), the maps promote routes travelling through the greenspace which allows children to be surrounded by nature (Mechanism), leading to promoted motivation to engage in active school travel (Outcome).

Preliminary CMOC 16 – 'Active Travel is not my Thing'

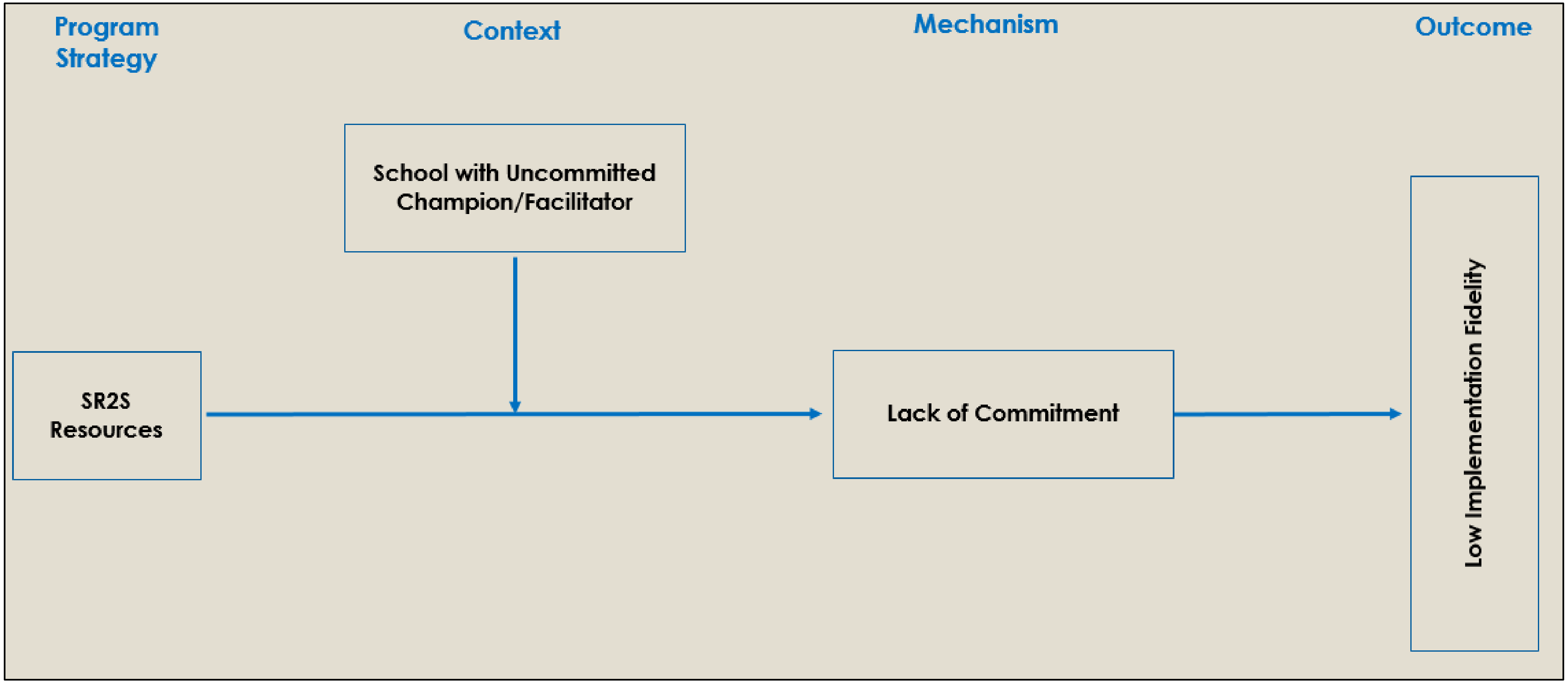

When the SR2S intervention resources (Strategy), are provided to a primary school that has an uncommitted active travel champion/facilitator (Context), the intervention strategies are not implemented/promoted effectively because the champion/facilitator lacks commitment to the program or active travel (Mechanism), leading to low implementation fidelity (Outcome).
