# Supplementary material for: Changing Primary School Children’s Engagement in Active School Travel Using Safe Routes to School Interventions: A Rapid Realist Review
Source: Int J Environ Res Public Health. 2022 Aug 12;19(16):9976. doi: 10.3390/ijerph19169976 (PMC9408451; doi:10.3390/ijerph19169976)
Supplement: Supplementary file 1 [file ijerph-19-09976-s001.zip › Supplementary_File_S2.pdf]

Search One

Database Name: Scopus			
Date of Search: 26/03/2020			
Field Codes: Title, Abstract, and Keywords (TITLE-ABS-KEY)			
Search #	Concept	Search Terms	No. of Results
#1	Active Travel	Walking OR Cycling OR Bicycling OR “Active Transport*” OR “Active School Transport*” OR “Active School Travel” OR “Active* Travel*” OR Commut* OR “Independent* Mobil*” OR “Independent* Travel*” OR “Independent Licence*"	515,402
#2	Intervention	“Safe Routes to School” OR “Safe Routes” OR “SRTS” OR “SR2S”	2,091
#3	Population	Child* OR “School Child*” OR “Elementary School*” OR “Primary School*” OR Boys OR Girls OR Youth*	3,576,701
#4		#1 AND #2 AND #3	121

Database Name: Scopus			
Date of Search: 14/09/2021 (2021 Update)			
Field Codes: Title, Abstract, and Keywords (TITLE-ABS-KEY)			
Search #	Concept	Search Terms	No. of Results
#1	Active Travel	Walking OR Cycling OR Bicycling OR “Active Transport*” OR “Active School Transport*” OR “Active School Travel” OR “Active* Travel*” OR Commut* OR “Independent* Mobil*” OR “Independent* Travel*” OR “Independent Licence*"	38,071
#2	Intervention	“Safe Routes to School” OR “Safe Routes” OR “SRTS” OR “SR2S”	165
#3	Population	Child* OR “School Child*” OR “Elementary School*” OR “Primary School*” OR Boys OR Girls OR Youth*	183,567
#4		#1 AND #2 AND #3	9

Search One

Database Name: Web of Science			
Date of Search: 26/03/2020			
Field Codes: Topic (TS)			
Search #	Concept	Search Terms	No. of Results
#1	Active Travel	Walking OR Cycling OR Bicycling OR “Active Transport*” OR “Active School Transport*” OR “Active School Travel” OR “Active* Travel*” OR Commut* OR “Independent* Mobil*” OR “Independent* Travel*” OR “Independent Licence*"	1,809,389
#2	Intervention	“Safe Routes to School” OR “Safe Routes” OR “SRTS” OR “SR2S”	1,430
#3	Population	Child* OR “School Child*” OR “Elementary School*” OR “Primary School*” OR Boys OR Girls OR Youth*	2,217,658
#4		#1 AND #2 AND #3	162

Database Name: Web of Science			
Date of Search: 14/09/2021 (2021 Update)			
Field Codes: Topic (TS)			
Search #	Concept	Search Terms	No. of Results
#1	Active Travel	Walking OR Cycling OR Bicycling OR “Active Transport*” OR “Active School Transport*” OR “Active School Travel” OR “Active* Travel*” OR Commut* OR “Independent* Mobil*” OR “Independent* Travel*” OR “Independent Licence*"	98,211
#2	Intervention	“Safe Routes to School” OR “Safe Routes” OR “SRTS” OR “SR2S”	70
#3	Population	Child* OR “School Child*” OR “Elementary School*” OR “Primary School*” OR Boys OR Girls OR Youth*	112,181
#4		#1 AND #2 AND #3	9

Search One

Database Name: APA PsycInfo (via EBSCOhost)			
Date of Search: 26/03/2020			
Field Codes: Title, Abstract, and Keywords (TI, AB, and KW)			
Search #	Concept	Search Terms	No. of Results
#1	Active Travel	Walking OR Cycling OR Bicycling OR “Active Transport*” OR “Active School Transport*” OR “Active School Travel” OR “Active* Travel*” OR Commut* OR “Independent* Mobil*” OR “Independent* Travel*” OR “Independent Licence*"	24,041
#2	Intervention	“Safe Routes to School” OR “Safe Routes” OR “SRTS” OR “SR2S”	305
#3	Population	Child* OR “School Child*” OR “Elementary School*” OR “Primary School*” OR Boys OR Girls OR Youth*	810,392
#4		#1 AND #2 AND #3	27

Database Name: APA PsycInfo			
Date of Search: 14/09/2021 (2021 Update)			
Field Codes: Title, Abstract, and Keywords (TI, AB, and KW)			
Search #	Concept	Search Terms	No. of Results
#1	Active Travel	Walking OR Cycling OR Bicycling OR “Active Transport*” OR “Active School Transport*” OR “Active School Travel” OR “Active* Travel*” OR Commut* OR “Independent* Mobil*” OR “Independent* Travel*” OR “Independent Licence*"	791
#2	Intervention	“Safe Routes to School” OR “Safe Routes” OR “SRTS” OR “SR2S”	309
#3	Population	Child* OR “School Child*” OR “Elementary School*” OR “Primary School*” OR Boys OR Girls OR Youth*	888,235
#4		#1 AND #2 AND #3	0

Search One

Database Name: CINAHL PLUS (via EBSCOhost)			
Date of Search: 26/03/2020			
Field Codes: Title and Abstract (TI and AB)			
Search #	Concept	Search Terms	No. of Results
#1	Active Travel	Walking OR Cycling OR Bicycling OR "Active Transport*" OR "Active School Transport*" OR "Active School Travel" OR "Active* Travel*" OR Commut* OR "Independent* Mobil*" OR "Independent* Travel*" OR "Independent Licence*"	40,740
#2	Intervention	"Safe Routes to School" OR "Safe Routes" OR "SRTS" OR "SR2S"	256
#3	Population	Child* OR "School Child*" OR "Elementary School*" OR "Primary School*" OR Boys OR Girls OR Youth*	589,255
#4		#1 AND #2 AND #3	22

Database Name: CINAHL			
Date of Search: 14/09/2021 (2021 Update)			
Field Codes: Title and Abstract (TI and AB)			
Search #	Concept	Search Terms	No. of Results
#1	Active Travel	Walking OR Cycling OR Bicycling OR "Active Transport*" OR "Active School Transport*" OR "Active School Travel" OR "Active* Travel*" OR Commut* OR "Independent* Mobil*" OR "Independent* Travel*" OR "Independent Licence*"	4,851
#2	Intervention	"Safe Routes to School" OR "Safe Routes" OR "SRTS" OR "SR2S"	29
#3	Population	Child* OR "School Child*" OR "Elementary School*" OR "Primary School*" OR Boys OR Girls OR Youth*	66,589
#4		#1 AND #2 AND #3	1

Search Two

Database Name: Scopus			
Date of Search: 31/03/2020			
Field Codes: Title & Abstract (TITLE-ABS)			
Search #	Concept	Search Terms	No. of Results
#1	Active Travel	Walking OR Cycling OR Bicycling OR “Active Transport*” OR “Active School Transport*” OR “Active School Travel” OR “Active* Travel*” OR Commut* OR “Independent* Mobil*” OR “Independent* Travel*” OR “Independent Licence*" OR “Crossing Guard*” OR “Crossing Supervisor” OR “Walking School Bus*” OR “School Travel Plan*” OR “Safe Routes to School”	394,353
#2	Population	Child* OR “School Child*” OR “Elementary School*” OR “Primary School*” OR Boys OR Girls OR Youth*	2,272,688
#3	Qualitative	Qualitative OR Interview* OR “Focus Group*”	1,201,337
#4		#1 AND #2 AND #3	721

Database Name: Scopus			
Date of Search: 14/09/2021 (2021 Update)			
Field Codes: Title & Abstract (TITLE-ABS)			
Search #	Concept	Search Terms	No. of Results
#1	Active Travel	Walking OR Cycling OR Bicycling OR “Active Transport*” OR “Active School Transport*” OR “Active School Travel” OR “Active* Travel*” OR Commut* OR “Independent* Mobil*” OR “Independent* Travel*” OR “Independent Licence*" OR “Crossing Guard*” OR “Crossing Supervisor” OR “Walking School Bus*” OR “School Travel Plan*” OR “Safe Routes to School”	38,088
#2	Population	Child* OR “School Child*” OR “Elementary School*” OR “Primary School*” OR Boys OR Girls OR Youth*	183,680
#3	Qualitative	Qualitative OR Interview* OR “Focus Group*”	134,727
#4		#1 AND #2 AND #3	126

Search Two

Database Name: Web of Science Core Collection			
Date of Search: 31/03/2020			
Field Codes: Topic (TS)			
Search #	Concept	Search Terms	No. of Results
#1	Active Travel	Walking OR Cycling OR Bicycling OR “Active Transport*” OR “Active School Transport*” OR “Active School Travel” OR “Active* Travel*” OR Commut* OR “Independent* Mobil*” OR “Independent* Travel*” OR “Independent Licence*" OR “Crossing Guard*” OR “Crossing Supervisor” OR “Walking School Bus*” OR “School Travel Plan*” OR “Safe Routes to School”	1,556,662
#2	Population	Child* OR “School Child*” OR “Elementary School*” OR “Primary School*” OR Boys OR Girls OR Youth*	1,842,385
#3	Qualitative	Qualitative OR Interview* OR “Focus Group*”	827,578
#4		#1 AND #2 AND #3	2,072

Database Name: Web of Science Core Collection			
Date of Search: 14/09/2021 (2021 Update)			
Field Codes: Topic (TS)			
Search #	Concept	Search Terms	No. of Results
#1	Active Travel	Walking OR Cycling OR Bicycling OR “Active Transport*” OR “Active School Transport*” OR “Active School Travel” OR “Active* Travel*” OR Commut* OR “Independent* Mobil*” OR “Independent* Travel*” OR “Independent Licence*" OR “Crossing Guard*” OR “Crossing Supervisor” OR “Walking School Bus*” OR “School Travel Plan*” OR “Safe Routes to School”	186,572
#2	Population	Child* OR “School Child*” OR “Elementary School*” OR “Primary School*” OR Boys OR Girls OR Youth*	217,783
#3	Qualitative	Qualitative OR Interview* OR “Focus Group*”	142,499
#4		#1 AND #2 AND #3	438

Search Two

Database Name: APA PsycInfo (via EBSCOhost/English Only)			
Date of Search: 31/03/2020			
Field Codes: Title (TI) & Abstract (AB)			
Search #	Concept	Search Terms	No. of Results
#1	Active Travel	Walking OR Cycling OR Bicycling OR “Active Transport*” OR “Active School Transport*” OR “Active School Travel” OR “Active* Travel*” OR Commut* OR “Independent* Mobil*” OR “Independent* Travel*” OR “Independent Licence*" OR “Crossing Guard*” OR “Crossing Supervisor” OR “Walking School Bus*” OR “School Travel Plan*” OR “Safe Routes to School”	21,519
#2	Population	Child* OR “School Child*” OR “Elementary School*” OR “Primary School*” OR Boys OR Girls OR Youth*	740,905
#3	Qualitative	Qualitative OR Interview* OR “Focus Group*”	393,147
#4		#1 AND #2 AND #3	293

Database Name: APA PsycInfo (via EBSCOhost/English Only)			
Date of Search: 14/09/2021 (2021 Update)			
Field Codes: Title (TI) & Abstract (AB)			
Search #	Concept	Search Terms	No. of Results
#1	Active Travel	Walking OR Cycling OR Bicycling OR “Active Transport*” OR “Active School Transport*” OR “Active School Travel” OR “Active* Travel*” OR Commut* OR “Independent* Mobil*” OR “Independent* Travel*” OR “Independent Licence*" OR “Crossing Guard*” OR “Crossing Supervisor” OR “Walking School Bus*” OR “School Travel Plan*” OR “Safe Routes to School”	1,711
#2	Population	Child* OR “School Child*” OR “Elementary School*” OR “Primary School*” OR Boys OR Girls OR Youth*	42,625
#3	Qualitative	Qualitative OR Interview* OR “Focus Group*”	32,742
#4		#1 AND #2 AND #3	33

Search Two

Database Name: CINAHL PLUS w/ Full Text (via EBSCOhost/English Only)			
Date of Search: 31/03/2020			
Field Codes: Title (TI) & Abstract (AB)			
Search #	Concept	Search Terms	No. of Results
#1	Active Travel	Walking OR Cycling OR Bicycling OR “Active Transport*” OR “Active School Transport*” OR “Active School Travel” OR “Active* Travel*” OR Commut* OR “Independent* Mobil*” OR “Independent* Travel*” OR “Independent Licence*" OR “Crossing Guard*” OR “Crossing Supervisor” OR “Walking School Bus*” OR “School Travel Plan*” OR “Safe Routes to School”	40,203
#2	Population	Child* OR “School Child*” OR “Elementary School*” OR “Primary School*” OR Boys OR Girls OR Youth*	572,058
#3	Qualitative	Qualitative OR Interview* OR “Focus Group*”	293,845
#4		#1 AND #2 AND #3	268

Database Name: CINAHL PLUS w/ Full Text (via EBSCOhost/English Only)			
Date of Search: 14/09/2021 (2021 Update)			
Field Codes: Title (TI) & Abstract (AB)			
Search #	Concept	Search Terms	No. of Results
#1	Active Travel	Walking OR Cycling OR Bicycling OR “Active Transport*” OR “Active School Transport*” OR “Active School Travel” OR “Active* Travel*” OR Commut* OR “Independent* Mobil*” OR “Independent* Travel*” OR “Independent Licence*" OR “Crossing Guard*” OR “Crossing Supervisor” OR “Walking School Bus*” OR “School Travel Plan*” OR “Safe Routes to School”	4,851
#2	Population	Child* OR “School Child*” OR “Elementary School*” OR “Primary School*” OR Boys OR Girls OR Youth*	66,589
#3	Qualitative	Qualitative OR Interview* OR “Focus Group*”	37,752
#4		#1 AND #2 AND #3	31
