# Supplementary material for: Changing Primary School Children’s Engagement in Active School Travel Using Safe Routes to School Interventions: A Rapid Realist Review
Source: Int J Environ Res Public Health. 2022 Aug 12;19(16):9976. doi: 10.3390/ijerph19169976 (PMC9408451; doi:10.3390/ijerph19169976)
Supplement: Supplementary file 1 [file ijerph-19-09976-s001.zip › Supplementary_File_S3.pdf]

| Reference | Author | Publication Year | Document Type | Methodology | Document Type | Intervention Type | Country | Study Aim/Purpose | Theory, Model, or Framework | Study Setting | Participants | Socio-demographic Factors | Data Collection Methods | Analytic Approach | Summary of Findings |
|---|---|---|---|---|---|---|---|---|---|---|---|---|---|---|---|
| [37] | Buttazzoni, Coen & Gilliland | 2018 | Research Article | Qualitative | Intervention | SR2S | CAN | To evaluate the factors shaping the functioning of STP interventions at ten elementary schools participating in a regional ASR2S program in Southwestern Ontario. | Kurt Lewin's Theory of Organisational Change | Elementary schools (*n*=10)<br><br>Elgin-St. Thomas<br>London<br>Middlesex<br>Oxford<br><br>Southwestern Ontario | Parents (*n*=7)<br><br>STP individuals (*n*=33)<br><br>Public health nurse (*n*=12)<br><br>Principal (*n*=7)<br><br>Teachers (*n*=2)<br><br>Community partner (*n*=1)<br><br>Town/city representatives (*n*=3) | Not reported | Semi-structured interviews (*n*=18)<br><br>Focus groups (*n*=4) | Thematic analysis | This study found six themes that demonstrated STP success is underpinned by a diversity of factors that range in scope from operational (e.g., meeting structure) to cultural (e.g., buy-in). This makes clear that in conceptualizing a plan for STP success, it is just as important that seemingly mundane aspects of committee operations be given just as serious consideration as is taking into account the specificities and needs of the local school context. It is suggested that public health and community interventions aimed to support AST should i) emphasize the importance of thorough pre-implementation assessments and build 'active school environments', and ii) foster the development of a collaborative approach, a robust operational framework or schedule, and a school-wide pro-AST culture. |
| [38] | Atteberry *et al.* | 2016 | Research Article | Qualitative | Intervention | SR2S | USA | To present an in-depth understanding of SR2S implementation in Texas, including the grant planning process, implementation, and future plans for the SR2S program in the local communities. | Not reported | Elementary schools (*n*=79)<br><br>Infrastructure schools (*n*=23)<br>non-infrastructure schools (*n*=22)<br>Comparison schools (*n*=34)<br><br>Texas, USA | Community members (*n*=31)<br><br>City officials (*n*=13)<br><br>Community organisations (*n*=8)<br><br>Elected official (*n*=1)<br><br>Public works employees (*n*=4)<br><br>School and district administrators (*n*=5) | Not reported | Semi-structured telephone interviews (*n*=31) | Thematic analysis | This study found community representatives reported accomplishments toward planned goals (improved infrastructure and perceived increase in active commuting), many had significant challenges including lack of communication and up-front funding, and difficulty navigating the regulatory process. Future SR2S programs should be structured to be more compatible with community-based needs and limitations, provide adequate underlying infrastructure and resources, and include at least partial funding up-front. |
| [39] | Crawford & Garrard | 2013 | Research Article | Mixed method | Intervention | SR2S + other | AUS | To evaluate the impact of the Ride2School program in a number of school communities. | Community-based Social Marketing Model | <u>Pilot phase</u><br>Intervention school (*n*=2)<br>Comparison school (*n*=2)<br><br>Inner suburban (*n*=2)<br>Outer suburban (*n*=2)<br><br><u>Program Phase</u><br>Program schools (*n*=13)<br><br>Rural (*n*=9)<br>Urban (*n*=4)<br><br>Victoria | <u>Pilot phase</u><br>Ride2School coordinator (*n*=1)<br><br><u>Program phase</u><br>School principal (*n*=11)<br>Teacher (*n*=21)<br>Student (*n*=70)<br>Ride2School coordinator (*n*=2)<br>Parents (*n*= 768)<br><br><u>Baseline (*n*=410)</u><br><br>Female (*n*=365)<br>Male (*n*=45)<br><br>18-29 years (*n*=11)<br>30-39 years (*n*=150)<br>40-49 years (*n*=223)<br>≥ 50 years (*n*=24)<br><br>Born Australia (*n*=347)<br>Born overseas (*n*=63)<br><br><u>Follow-up (*n*=358)</u><br>Female (*n*=315)<br>Male (*n*=39)<br><br>18-29 years (*n*=3)<br>30-39 years (*n*=118)<br>40-49 years (*n*=205)<br>≥ 50 years (*n*=30)<br><br>Born Australia (*n*=299)<br>Born overseas (*n*=58)<br><br>Children (*n*=882)<br>Baseline (*n*=479)<br><br>Female (*n*=253)<br>Male (*n*=226)<br><br>Grade 4 (*n*=140)<br>Grade 5 (*n*=166)<br>Grade 6 (*n*=173)<br><br>Follow-up (*n*=403)<br><br>Female (*n*=207)<br>Male (*n*=196)<br><br>Grade 4 (*n*=133)<br>Grade 5 (*n*=138)<br>Grade 6 (*n*=123) | <u>Program phase</u><br><br><u>Parent education</u><br><u>Baseline</u><br>Bachelor or higher (*n*=112)<br>Diploma (*n*=48)<br>Vocation (*n*=92)<br>No qualification (*n*=143)<br><br><u>Follow-up</u><br>Bachelor or higher (*n*=117)<br>Diploma (*n*=46)<br>Vocation (*n*=53)<br>No qualification (*n*=113) | <u>Pilot phase</u><br><u>Quantitative</u><br>Observational route counts<br><br>Hands up surveys<br><br><u>Qualitative</u><br>Semi structured interviews (*n*=1)<br><br><u>Program phase</u><br><u>Quantitative</u><br>Parent/student survey<br><br><u>Qualitative</u><br>Semi structured telephone interviews (*n*=not reported)<br><br>Focus groups (*n*=not reported) | <u>Quantitative</u><br>Descriptive analysis<br><br>Comparative analysis<br><br>Logistic regression modelling<br><br><u>Qualitative</u><br>Thematic analysis | This study found mixed evidence for the effectiveness of the Ride2School program. Relatively intensive support for active transport to school, supportive environment for active transport, and strong school commitment to promoting active transport, appeared to be effective in increasing active transport to school. Less intensive support and resourcing was less effective in increasing active transport to school, though a small number of schools achieved small-to-modest increases in active transport to school. |
| [40] | McDonald *et al.* | 2014 | Research Article | Quantitative | Intervention | SR2S | USA | To assess the impacts of federally funded SR2S programs on walking and bicycling across four states | Not reported | States (*n*=4)<br><br>Florida<br>Oregon<br>Washington DC<br>Texas | School (*n*=801)<br><br>Intervention schools (*n*=378)<br>Control schools (*n*=423) | <u>Free/reduced price lunch</u><br>Intervention schools (61%)<br>Control schools (61%)<br><br><u>Median household income</u> | Secondary data set (national Centre for SR2S) | Fractional logit modelling | This study found SR2S programs has demonstrated significant increases in walking and bicycling. Analysis of data from 801 schools in DC, Florida, Oregon, and Texas indicates an absolute increase of |

| | | | | | | | | | | | | | | | |
|---|---|---|---|---|---|---|---|---|---|---|---|---|---|---|---|
| | | | | | | | | | | | Elementary schools (83%)<br><br>Black (14%)<br>Hispanic (42%)<br>Mixed race (3%)<br>White (38%) | Intervention ($53,074)<br>Control ($50,550) | | | 5.5 percentage points or a relative change of 31% in the proportion of students walking and bicycling to school after five years of participating in a SR2S program. |
| [41] | McDonald *et al.* | 2013 | Research Article | Quantitative | Intervention | SR2S | USA | To measure the impacts of Eugene, Oregon's Safe Routes to School program on walking and biking. | Not reported | Schools (*n*=14)<br><br>Intervention (*n*=9)<br>Control (*n*=5)<br><br>Elementary (*n*=10)<br>Middle (*n*=3)<br>K-8 (*n*=1)<br><br>Eugene<br><br>Oregon | Intervention schools (*n*=9)<br>American-Indian (1.2%)<br>Asian/Pacific islander (3.8%)<br>Black (2.8%)<br>Hispanic (14.3%)<br>Multi-racial (8.5%)<br>White (69.4%)<br><br>Control schools (*n*=5)<br>American-Indian (1.5%)<br>Asian/Pacific islander (2.7%)<br>Black (3.1%)<br>Hispanic (15.6%)<br>Multi-racial (8.5%)<br>White (68.5%) | Free/reduced price lunch<br>Intervention schools (44.8%)<br>Control schools (48.2)<br><br>Achievement Index<br>Intervention schools (89.8%)<br>Control schools (89.5%) | Secondary data set (national Centre for SR2S) | Fractional logit modelling | This study found that Eugene's Safe Routes to School program has increased walking and biking as school transport modes. Education and encouragement programs were associated with a five-percentage point increase in biking. Walking and biking increased most when schools implemented multiple SR2S interventions. |
| [42] | Mammen *et al.* | 2015 | Research Article | Qualitative | Intervention | STP | CAN | To identify facilitators and barriers to effective School Travel Planning (STP) implementation. | Not reported | Provinces (*n*=3)<br><br>Ontario<br>West of Ontario<br>East of Ontario<br><br>Ontario | School Travel Plan facilitators (*n*=34)<br>Female (*n*=27)<br>Male (*n*=7)<br><br>Urban (*n*=3)<br>Suburban (*n*=31) | Not reported | Semi-structured telephone interviews (*n*=34) | Thematic analysis | This study found STP facilitators perceived the program to be successful, however, definitions of success differed among facilitators. Factors such as a well-designed STP model, active collaboration between multidisciplinary stakeholder and the facilitators leadership role were important for effective implementation. Lack of stakeholder involvement and the time given by funding bodies for program implementation were identified as the primary barriers. |
| [43] | Stewart *et al.* | 2014 | Research Article | Quantitative | Intervention | SR2S | USA | To assess changes in rates of AST after implementation of SR2S at multiple sites across four states. | Not reported | States (*n*=4)<br><br>Florida<br>Mississippi<br>Washington<br>Wisconsin | Schools (*n*=53)<br><br>Completed SR2S projects (*n*=48) | Not reported | Secondary data set (SR2S project tracking database) | Comparative analysis<br><br>Bivariate analysis | This study found walking increased by 45%, bicycling increased by 24%, and all AST increased by 37%. More comprehensive SR2S projects with a smaller reach tended to have greater increases in AST. For bicycling only, lower baseline rates of bicycling to school were significantly related to greater increases in rates of bicycling to school after implementation of an SR2S project. |
| [44] | Hinckson | 2016 | Research Article | Qualitative | Intervention | WSB | NZ | To explore the perceived challenges and facilitators of active travel to and from school following implementation of the STP Programme through qualitative feedback from children and adolescents. | Not reported | Schools (*n*=13)<br><br>Primary (*n*=10)<br>High (*n*=3)<br><br>Auckland | Children (*n*=52)<br><br>Adolescents (*n*=26) | Not reported | Focus groups (*n*= Not reported) | Thematic analysis | This study found challenges and facilitators for children and adolescents focused primarily on intrapersonal, and interpersonal factors. The themes emerged were similar but for different contexts. Under the child factors category five main themes emerged: peers, enjoyment, safety, parent behaviour and health and fitness. The main themes for adolescents were peers/enjoyment, driving licence, safety and health & engagement. |
| [45] | Savolainen *et al.* | 2020 | Research Article | Qualitative | Intervention | Gamification | SWE | To follow up on participants' experiences one and two years after the AST intervention was initiated. | Social Cognitive Theory | Primary school (*n*=1)<br><br>Northern Sweden | Children (*n*=71)<br><br>Teachers (*n*=2)<br><br>School principal (*n*=1) | School SES<br>Ranked among the 10% most socioeconomically privileged | Semi-structured interviews (*n*=1)<br><br>Focus groups (*n*=18) | Content analysis | This study found the concept of the intervention was attractive to re-use, both from the perspective of staff and pupils, the year after and two years after the first time. Participants developed a habit of using AST and that the intervention could therefore be a source of increased physical activity. Traffic around the school decreased, which could be a valuable incentive for schools to engage in the intervention. Interventions to promote AST can benefit from the use of engagement, togetherness, and gamification. |
| [46] | Teller *et al.* | 2019 | Research Article | Qualitative | Intervention | WSB | USA | To assess parent perceptions of a WSB program to inform future programs. | Not reported | Elementary schools (*n*=9)<br><br>Seattle | Parents (*n*=45)<br>Female (82%)<br>Male (18%)<br><br>27-35 (22%)<br>36-45 (45%)<br>46-60 (16%)<br>Not reported (9%)<br><br>Hispanic (22%)<br>Non-hispanic white (27%)<br>Non-hispanic black (9%)<br>Asian-american (20%)<br>Other/not reported (22%) | Parent education<br>High school or less (29%)<br>Some college/associates/vocational (36%)<br>Bachelor's/postgraduate (24%)<br>Not reported (11%)<br><br>Household income<br>≤$20,000 (22%)<br>$20,001–$40,000 (31%)<br>$40,001–$60,000 (24%)<br>>$60,000 (22%) | Semi-structed interviews (*n*=45) | Framework analysis | This study found that the perceived benefits extended far beyond physical health, with parents citing everything from their children learning pedestrian safety rules to gaining independence and self-confidence. Parents have identified several ways to improve WSB programs; in particular, when designing programs, researchers can use parent recommendations from this study to create more inclusive programs and streamline communication. |
| [47] | Nikitas, Wang & Knamiller | 2019 | Research Article | Qualitative | Intervention | WSB | UK | To develop a more in-depth understanding of the factors that define parents' travel mode choice for their children's daily transport to school and may prevent or motivate parents to commit to a WSB initiative. | Not reported | Primary Schools (*n*=2)<br><br>Bradford | Parents (*n*=25)<br><br>Female (*n*=23)<br>Male (*n*=2) | Not reported | Focus groups (*n*=4) | Thematic analysis | This study found that logistics, safety, trust, health & wellbeing, emotional needs and educational opportunities were key issues underpinning parental adoption of a WSB. |
| [48] | Race *et al.* | 2017 | Research Article | Qualitative | Non-intervention | N/A | CAN | To describe the school travel experience of children who live in a highly walkable, downtown urban setting and those who | Not reported | Elementary schools (*n*=3) | Children (*n*=42)<br><br>Boys (*n*=24) | Parent education<br>Did not complete high school (*n*=1) | Focus groups (*n*=9) | Framework analysis | This study found that children were able to provide valuable insight into their school travel |

| | | | | | | | | | | | | | | | |
|---|---|---|---|---|---|---|---|---|---|---|---|---|---|---|---|
| | | | | | | | | live in less walkable suburban neighbourhoods in Vancouver by identifying perceived barriers and facilitators to AST. | | Metropolitan Vancouver<br><br>British Columbia | Girls (*n*=18)<br><br>Urban (*n*=16)<br>Suburban (*n*=26)<br><br>Caucasian (*n*=12)<br>Asian (*n*=19)<br>Other (*n*=8)<br>Mixed (*n*=2)<br>Data missing (*n*=1)<br><br>English speaking (*n*=19)<br>Other than English (*n*=22)<br>Data missing (*n*=1) | Completed high school (*n*=10)<br>Some college or vocational (*n*=5)<br>College/university (*n*=16)<br>Graduate degree (*n*=4)<br>Missing data (*n*=6)<br><br><u>Household income</u><br>≥ $80,000 (*n*=13)<br>$40,000 - $79,999 (*n*=12)<br>≤ $39,999 (*n*=8)<br>Missing data (*n*=9) | | | experiences. Urban and suburban children were shown to experience similar barriers and facilitators to AST. However, the extent to which these factors influenced AST, unsurprisingly, varied between neighbourhood types and between individuals. Although many children overcame barriers such as personal safety to commute to/from school each day, we recommend that schools engage children in the design of AST programs. |
| [49] | Benson, Bruner & Mayer | 2020 | Research Article | Qualitative | Intervention | WSB | CAN | To explore the experiences of individuals involved with the pilot walking school bus program to provide insights into the strengths, weaknesses, and lessons learned from the School Travel Planning initiative in North-eastern Ontario. | Pragmatist Framework | Elementary schools (*n*=2)<br><br>North-eastern Ontario | Parents (*n*=7)<br><br>Children (*n*=18)<br><br>Conductors (*n*=2) | Not reported | Semi-structured telephone interviews (*n*=7)<br><br>Focus groups (*n*=3) | Thematic analysis | This study found key lessons for operating a WSB. WSB need to: increase community involvement and sense of community; use a diverse volunteer base (i.e., so not solely reliant on parent volunteers); provide a pedestrian education component; develop knowledge of safe routes to school; build community partnerships for success; and be implemented in consideration of the weather patterns in the area. |
| [50] | Chaufan *et al.* | 2012 | Research Article | Quantitative | Intervention | SR2S | USA | To evaluate SR2S state-level projects targeting students and kindergarten through to ninth grade using first phase (2008-2010) data from ongoing SR2S programs. | Not reported | Town and cities (*n*=81)<br><br>California | Schools (*n*=392)<br><br>Parents (*n*=63,078) | Not reported | Secondary data set (not reported) | Descriptive analysis | This study found modes of school transport among children and parental concerns regarding active commuting in California could conceivably be eased through appropriate interventions and the disproportionate percentage of children who are driven to school rather than walking or biking is salient. |
| [51] | Crawford *et al.* | 2017 | Research Article | Qualitative | Non-intervention | N/A | AUS | To explore the views and experiences of both children and parents in relation to children's independent mobility. | Social Ecological Framework | Schools (*n*=7)<br><br>Primary (*n*=5)<br>High (*n*=1)<br>P-12 (*n*=1)<br><br>Metropolitan (*n*=5)<br>Rural (*n*=2)<br><br>Victoria | <u>Parents (*n*=12)</u><br>Female (83.3%)<br>Male (16.7%)<br><br><u>Children (*n*=132)</u><br>Female (56.8%)<br>Male (43.2%) | Not reported | Focus groups (*n*=12) | Thematic analysis | This study found children's awareness of their parents' wishes and worries, and their delight in socializing with their peers independent of adults were evident. There exists a balancing act for parents, in which they seek to maintain family routines and cater for children's need for independence, while simultaneously ensuring their safety. Parents and children have somewhat different concerns about the risks associated CIM, and that parents' decisions around the freedoms they grant their children are shaped by their social context. |
| [52] | Zhou *et al.* | 2010 | Research Article | Quantitative | Intervention | SR2S | USA | To apply a new statistical diagnosis approach to conduct a sequential search of the necessary-sufficient significant factors for AST from multiple perspectives. | Not reported | Schools (*n*=18)<br><br>Elementary (*n*=16)<br>Middle (*n*=2)<br><br>Pinellas County<br><br>Florida | Parents (*n*=2551)<br><br>Classrooms (*n*=644) | Not reported | Walking and bicycling to school survey for parents<br><br>SR2S Student arrival and departure tally Sheet | Multiple perspectives analysis | This study provided deeper understanding of the barriers that result in the low walking/biking rates. Based on the multiple perspectives analysis results, safety and security remain the top concerns for parents who live within walkable distances. Parent's comments show that it will be nonsense to let children walk/bike alone to school without a safe community environment. Traffic safety–related issues are also very highly ranked factors. Most of parents pointed out intersection safety as one of their major concerns. Distance is another most significant factor and the largest barrier for the long-distance interval group. Lack of sidewalk access to schools is another safety concern. Paving more sidewalks around schools may help to improve the walking/biking safety. In addition, subjective factors (school attitudes, allowable grade, and student's attitudes) are also significant factors. |
| [53] | Forsberg *et al.* | 2020 | Research Article | Qualitative | Non-intervention | N/A | SWE | To explore parents' attitudes and beliefs towards AST in the northern part of Sweden. | Theory of Planned Behaviour | Primary schools (*n*=5)<br><br>Northern Sweden | Parents (*n*=20)<br><br>Female (*n*=16)<br>Male (*n*=4)<br><br>35-53 years (*n*=20) | <u>Parents education</u><br>University Degree (*n*=17)<br>Upper secondary (*n*=3) | Semi-structed interviews (*n*=20) | Content analysis | This study found seasonal changes affect parents' decisions, and this needs to be considered in order to facilitate AST in these regions. Parents' decisions were influenced by social norms, the view of children's competence as well as independence, and how the idea of parenting has changed through the generations. |
| [54] | Florida Department of Health | 2012 | Grey Literature Report | Mixed method | Intervention | SR2S + other | USA | To describe the outcomes of the Florida CPPW Safe Routes to School (SR2S) Walking School Bus (WSB) initiative, which fulfilled the physical activity component of the grant by encouraging elementary school students to walk to and from school. | Not reported | Elementary school (*n*=4)<br><br>Florida | <u>Quantitative</u><br>Classroom (*n*=not reported)<br><br><u>Qualitative</u><br>Coordinators (*n*=not reported) | Not reported | <u>Quantitative</u><br>Travel tally<br><br><u>Qualitative</u><br>Focus groups (*n*=not reported) | <u>Quantitative</u><br>Descriptive analysis<br><br><u>Qualitative</u><br>Thematic analysis | This report found key stakeholders must be involved in all stages of the initiative from planning to evaluation, local partnerships are critical in starting and sustaining WSB programs, placing coordinators in regions throughout Florida was a key factor in allowing Florida to exceed its objective, the SR2S Students Arrival and Departure Tally Sheets are an appropriate data source when assessing changes in the number of |

| | | | | | | | | | | | | | | | |
|---|---|---|---|---|---|---|---|---|---|---|---|---|---|---|---|
| | | | | | | | | | | | | | | | students walking or biking within a school but are more difficult to use when assessing state-wide change, a school district's School Health Advisory Council (SHAC) is an effective partner when working toward the adoption of a SR2S policy, and Interactive logs on a shared drive are an effective system for regional staff to use in reporting milestones. |
| [55] | Ginja *et al.* | 2019 | Research Article | Qualitative | Intervention | Incentive | UK | To test the feasibility of an incentive scheme to increase ATS in Year 5 children. | Not reported | Primary school (*n*=2)<br><br>Intervention (*n*=1)<br>Control (*n*=1)<br><br>Newcastle | <u>Children (*n*=9)</u><br>Intervention school (*n*=5)<br>Control school (*n*=4)<br><br>Female (*n*=3)<br>Male (*n*=6)<br><br>White British (*n*=7)<br>Black African (*n*=1)<br>Chinese (*n*=1)<br><br><u>Parents (*n*=9)</u><br>Intervention school (*n*=5)<br>Control school (*n*=4)<br><br>Female (*n*=8)<br>Male (*n*=1)<br><br>White British (*n*=7)<br>Black African (*n*=1)<br>Chinese (*n*=1)<br><br><u>Teachers (*n*=4)</u><br>Intervention school (*n*=2)<br>Control school (*n*=2)<br><br>Female (*n*=3)<br>Male (*n*=1)<br><br>White British (*n*=4)<br><br><u>School receptionists (*n*=3)</u><br>Intervention school (*n*=2)<br>Control school (*n*=1)<br><br>Female (*n*=2)<br>Male (*n*=1)<br><br>White British (*n*=3)<br><br><u>Head teachers (*n*=2)</u><br>Intervention school (*n*=1)<br>Control school (*n*=1)<br><br>Female (*n*=2)<br><br>2 White British (*n*=2)<br><br><u>Charity representatives (*n*=2)</u><br>2 Male (*n*=2)<br><br>White British (*n*=2) | <u>Parent education</u><br>Degree or higher (*n*=3)<br>GCSE (*n*=3)<br>A-levels (*n*=2)<br>None (*n*=1)<br>Not reported (*n*=20) | Semi-structed interviews (*n*=29) | Thematic analysis | This study found participants enjoyed taking part in the RIGHT TRACKS study and engaged with it. However, a number of issues were raised pertaining to recruitment, data collection, and use of incentives. Key recommendations included close collaboration with organisations already working in schools, considering a different range of incentives, and the possibility of running the scheme as a lunchtime activity. |
| [56] | Donnellan, Egli & Smith | 2020 | Research Article | Mixed method | Intervention | Infrastructure | NZ | To understand the factors related to travel mode to school from the perspectives of children, their parents and school representatives, through the evaluation of a shared path. | Kids-PoND Framework | Schools (*n*=4)<br><br>Primary (*n*=3)<br>Intermediate (*n*=1)<br><br>Whangarei district | <u>Children (*n*=59)</u><br>Female (*n*=41)<br>Male (*n*=18)<br><br>12 years (*n*=16)<br>11 years (*n*=3)<br>10 years (*n*=15<br>9 years (*n*=23)<br>8 years (*n*=2)<br><br><u>Parents (*n*=35)</u><br><br><u>School representatives (*n*=4)</u> | <u>School decile rank</u><br>Decile rank 6 (*n*=1)<br>Decile rank 4 (*n*=2)<br>Decile rank 2 (*n*=1) | Quantitative Hands up survey<br><br>Qualitative Focus groups (*n*= 13)<br>Semi-structured telephone interviews (*n*=35)<br>Semi-structured interviews (*n*=4) | <u>Quantitative</u><br>Descriptive analysis<br><br><u>Qualitative</u><br>Thematic analysis | This study found three out of four schools reported small but promising increases in the proportion of AST from pre to post shared path intervention. Understanding multiple perspectives from children, parents and school representatives' revealed a number of factors related to children's mode of travel to school. The fun and the enjoyable aspects of children's commute to school, aesthetics, accessibility, utility and safety of the shared path intervention were important factors. Promoting the benefits of the shared path to the wider community and encouraging people of all ages to utilise the path daily could contribute to a positive perception of safety for children walking, biking or scootering to school. |
| [57] | Rutberg & Lindqvist | 2019 | Research Article | Mixed method | Intervention | Gamification | SWE | To describe parents' attitudes to AST and to explore their experience when implementing interventions to promote it. | Social Cognitive Theory | Primary school (*n*=1)<br><br>Northern Sweden | <u>Quantitative</u><br>Parents (*n*=63)<br>Female (*n*=35)<br>Male (*n*=26)<br><br><u>Qualitative</u><br>Parents (*n*=44)<br>Female (*n*=26)<br>Male (*n*=18)<br><br>Swedish citizens (97%) | <u>Parent education</u><br>College/university (73%)<br>Upper secondary (27%) | Questionnaire<br><br><br>Open-ended questionnaire | Content analysis | This study shows how beneficial it is to seize children's enthusiasm and motivation to overcome parental hesitation with AST. However, parental concerns must be acknowledged as they are the gatekeepers of the children's use of AST, and it is valuable to empower parents when designing pertinent interventions. The idea of children being accompanied by other children prompted a shift in behaviour. Interventions to increase AST should target |

| | | | | | | | | | | | | | | | |
|---|---|---|---|---|---|---|---|---|---|---|---|---|---|---|---|
| | | | | | | | | | | | | | | | changing behaviour and parental self-efficacy in their children's ability to safely use active transport, instead of focusing on changing parental attitudes. |
| [58] | Rutberg & Lindqvist | 2018 | Research Article | Qualitative | Non-intervention | N/A | SWE | To explore the experiences of schoolchildren and teachers participating in an empowerment and gamification-inspired program to promote children's active school transportation. | Social Cognitive Theory | Primary school (*n*=1)<br><br>Northern Sweden | Children (*n*=32)<br>Boys (*n*=17)<br>Girls (*n*=15)<br><br>Teachers (*n*=2)<br>Female (*n*=2) | Not reported | Focus groups (*n*=8) | Content analysis | This study found that that using gamification to combine learning with physical activity outside of school time is a promising method for promoting AST. Enriched learning activities created additional benefits beyond physical activity, such as a sense of togetherness and improved readiness to learn when the students arrive at school. |
| [59] | Lindqvist *et al.* | 2019 | Research Article | Qualitative | Non-intervention | N/A | SWE | To explore the prerequisites and experiences of schoolchildren and parents participating in an empowerment and gamification-inspired intervention to promote students' AST in winter conditions. | Social Cognitive Theory | Primary school (*n*=1)<br><br>Northern Sweden | Children (*n*=35)<br><br>Girls (*n*=20)<br>Boys (*n*=15)<br><br>Parents (*n*=34)<br><br>Female (*n*=22)<br>Male (*n*=12)<br><br>39-56 years (*n*=34)<br><br>Born in Sweden (*n*=31) | Parents education<br>University education (*n*=30) | Photovoice<br><br>Open-ended questionnaire | Content analysis | This study found interventions for increasing students' AST in winter conditions should focus on the motivational aspects for both children and parents instead of the physical environment. Empowerment and including the end-users throughout the project are key contributors to success and makes it possible to design a sustainable program. Targeting a change in behaviour instead of focusing on changing attitudes could be beneficial in overcoming parental hesitation with AST during winter conditions. |
| [60] | Ahern *et al.* | 2017 | Research Article | Qualitative | Non-intervention | N/A | UK | To explore parents' experiences of school travel and their choices regarding travel mode with a focus on identifying barriers and facilitators to active school travel. | Theoretical Domains Framework | Primary schools (*n*=6)<br><br>West Yorkshire | Parents (*n*=20)<br><br>Female (*n*=18)<br>Male (*n*=2)<br><br>White British (*n*=10)<br>Pakistani (*n*=7)<br>Indian (*n*=1)<br>Mixed (*n*=2) | Parents living in SES areas<br>Most deprived IMD=1 (*n*=10)<br><br>More deprived IMD=2-4 (*n*=7)<br><br>Average deprivation IMD=5 (*n*=1)<br><br>Less deprived IMD=6 (*n*=1)<br><br>Least deprived IMD-10 (*n*=1) | Semi-structured telephone interviews (*n*=18)<br><br>Focus groups (*n*=2) | Framework analysis | This study found distance to be the biggest barrier to AST. Time constraints were reported as the main barrier to parents accompanying their children. Concerns about safety deterred parents from allowing children to travel independently. The need to incorporate multiple journeys, such as the work commute and/or multiple school drop-offs, placed demands on parents time, while difficulty getting children into local schools meant further to travel for a number of parents. |
| [61] | Ahlport *et al.* | 2008 | Research Article | Qualitative | Non-intervention | N/A | USA | To describe the barriers and facilitators of walking and biking to school as reported by parents and children who live within a 1.5-mile radius of their school. | Social Ecological Framework<br><br>Political Economy of Health Framework | Elementary schools (*n*=4)<br><br>North Carolina | Parents (*n*=37)<br><br>Children (*n*=37)<br><br>Parent/child parings (*n*=37)<br><br>AT pairs (*n*=26)<br>Non-AST pairs (*n*=11) | Not reported | Focus groups (*n*=12) | Thematic analysis | This study found that the barriers and facilitators to AST reported by parents and children generally fell into three categories: intrapersonal and interpersonal characteristics of parents and children, environmental characteristics of the neighbourhood, and environmental and policy characteristics of the school. A supportive environment is a necessary but insufficient condition to increase walking and biking to school. Initiatives to increase active school travel may need to include multiple levels of intervention to be effective |
| [62] | Faulkner *et al.* | 2010 | Research Article | Qualitative | Non-intervention | N/A | CAN | To explore parental accounts of the school travel mode choice decision-making process. | Behavioural Economic Theory | Elementary school (*n*=4)<br><br>Toronto | Parents (*n*=27)<br><br>Female (*n*=30)<br>Male (*n*=7)<br><br>AST (*n*=17)<br>Non-AST (*n*=20) | School SES<br>Low (*n*=2)<br>High (*n*=2) | Semi-structed interviews (*n*=37) | Thematic analysis | This study found the trip to/from school was not important to the decision-making process of the parents in this study. Rather, travel to/from school involves a two-step parental decision-making process and these choices are influenced by related but different factors. While escort decisions are dictated by road/traffic and personal safety concerns, the behavioural cost and reinforcing value of travel mode alternatives dictate mode choice. |
| [63] | Francis *et al.* | 2017 | Research Article | Qualitative | Non-intervention | N/A | AUS | To explore parents' perceived risk of, and fear of, stranger danger; physical and social environmental factors influencing parents' fear, including differences by SES; and strategies to manage parents' fear. | Social Ecological Framework | Multiple suburbs (*n*=not reported)<br><br>Metropolitan Perth<br><br>Western Australia | Parents (*n*=33)<br>Grandparents (*n*=1)<br><br>Female (*n*=34)<br><br>20-24 (6%)<br>25-29 (9%)<br>30-34 (18%)<br>35-39 (32%)<br>40-44 (26%)<br>45-49 (6%)<br>50+ (3%)<br><br>Single (15%)<br>Married (70%)<br>De-facto (15%)<br><br>1 children (9%)<br>2 children (50%)<br>3 children (23.5%)<br>4 children (11.5%)<br>5 children (6%)<br><br>Male children (50%)<br>Female children (50%) | Suburb SES<br>Low (*n*=13)<br>Mid (*n*=12)<br>High (*n*=9)<br><br>Parent employment<br>Part-time (44%)<br>Full time (3%)<br>Self-employed (3%)<br>Maternity leave (3%)<br>Unemployed (47%) | Focus groups (*n*=7) | Content analysis | This study found many competing perspectives and experiences are framed around the concept of keeping children safe. For many parents, the fear of strangers is real, and the consideration that people they know could be more likely to hurt their child aggravates rather than alleviates this fear. Strategies to allay parents' fear for their children's safety need to address both perceptions of fear and physical and social environment elements of the neighbourhood. |
| [64] | Greves *et al.* | 2007 | Research Article | Qualitative | Non-intervention | N/A | USA | To examine beliefs and barriers among immigrant families for walking to school and school breakfast participation in | Social Ecological Framework | Low income neighbourhoods (*n*=not reported) | Parents and grandparents (*n*=53)<br><br>18 Spanish (*n*=18) | Parent education<br>Grade school or less (45%)<br>High school degree (32%)<br>College degree (10%) | Focus groups (*n*=6) | Thematic analysis | This study found that immigrant families residing in low-income neighbourhoods largely supported the ideas of implementing a WSB. |

| | | | | | | | | | | | | | | | |
|---|---|---|---|---|---|---|---|---|---|---|---|---|---|---|---|
| | | | | | | | | order to guide development of a school-based obesity prevention program. | | Seattle<br><br>Washington | 18 Somali (*n*=18)<br>17 Vietnamese (*n*=17)<br><br>Female (86%)<br>Male (14%) | | | | However, major barriers were identified at the community, institutional and built environmental levels. Program such as these need to firstly address these barriers before parents fully engage in a WSB program. |
| [65] | O'Connor & Brown | 2013 | Research Article | Qualitative | Non-intervention | N/A | AUS | To explore how fear might be comprehended as something more than a reduction of public anxieties about crime generated through numbers on a survey, particularly if we are to better understand it's impact upon health related and other behaviour. | Parameters of Fear Framework | Primary school (*n*=1)<br><br>Melbourne<br><br>Victoria | Parents (*n*=24)<br><br>Female (*n*=24) | Advantaged end of the social disadvantage scale | Semi-structured interviews (*n*=not reported)<br><br>Focus groups (*n*=not reported) | Not reported | This study found fear in relation to IAST is not underpinned by a singular powerful 'globalised' narrative, but is patterned, practiced and emerges from the way individuals engage with their world. The extent to which individuals, through everyday interactions, make sense of and develop different notions of fear is also important. Fear is the product of interlocking and dynamic relations between broad environmental, socio-cultural, and temporal drivers of fear entwined with personal accounts, bodily experiences and social interactions. |
| [66] | Jago *et al.* | 2009 | Research Article | Qualitative | Non-intervention | N/A | UK | To examine parental attitudes to independent activity, factors that limit licence to be independently active and parental strategies to facilitate independent activity. | Not reported | Primary schools (*n*=6)<br><br>Bristol | Parents (*n*=24)<br><br>Female (*n*=20)<br>Male (*n*=4) | <u>Parents by school SES</u><br>Low (*n*=12)<br>Middle(*n*=9)<br>High (*n*=3) | Semi-structured telephone interviews (*n*=24) | Thematic analysis | This study found children's options to be active on their own are limited by safety concerns, a perceived lack of an appropriate space, the proximity of friends and traffic. Parents indicated that the end of primary school is a period when they begin to afford their children increased licence to be active and manage activity. Strategies that manage parental concerns either indirectly through reducing risk in the local environment or directly through enhanced parental licence are important to promote increased independent physical activity in young people. |
| [67] | Heinrich *et al.* | 2011 | Research Article | Quantitative | Intervention | SR2S | USA | To determine baseline rates for active transportation rates to and from school and to track changes related to macro-level (state-wide) policy, locally-based Safe Routes to School (SR2S) programs and bicycle and pedestrian planning initiatives expected to improve the safety, comfort and ease of walking and bicycling to and from school. | Not reported | Schools (*n*=13)<br><br>Intervention schools (*n*=8)<br>Comparison schools (*n*=5)<br><br>Rural (*n*=5)<br>Urban (*n*=8)<br><br>Asian, native Hawaiian, pacific islander<br>Intervention (60.3%)<br>Comparison (72.9%)<br><br>Hawaii | <u>Parent (*n*=1648)</u><br>Intervention (*n*=1191)<br>Comparison (*n*=457)<br><br><u>Classrooms</u><br>Interventions (*n*-40)<br>Comparison (*n*=13) | <u>Free and reduced school lunch</u><br>Intervention schools (51.5%)<br>Comparison schools (73.9%)<br><br><u>Parents graduated college</u><br>Intervention schools (25.0%)<br>Comparison schools (13.8%) | Parent survey<br><br>Student travel tally<br><br>Traffic counts and safety observations<br><br>Pedestrian Environment Audit (PEDS) | Descriptive analysis | This study found it is important to address traffic problems and missing infrastructure in order to increase walking and biking to school by low-income. Project collaborations should utilize these results to move forward necessary changes in programming, policies, and the physical environment. |
| [68] | Henderson *et al.* | 2013 | Research Article | Quantitative | Intervention | SR2S | USA | To report the findings associated with the successful implementation of a SR2S program and identify elements of the program that led to its success. | Not reported | Elementary School (*n*=1)<br><br>Metropolitan Atlanta<br><br>Georgia | Parents (*n*=not reported) | Not reported | Parent survey | Descriptive analysis | This study found behaviour change at the community level is quite complex and involves massive and coordinated efforts among multiple sectors of a community (e.g., schoolteachers, police officers, urban planners) who often may not be considered traditional public health partners. In addition, an informed and engaged community that participates on its own behalf is the foundation of human and community capacity-building. |
| [69] | Hoelscher *et al.* | 2016 | Research Article | Quantitative | Intervention | SR2S | USA | To determine the effects of differing funding allocation methods (infrastructure vs. non infrastructure) on student ACS, student physical activity and psychosocial antecedents, and parent ACS-related psychosocial constructs and behaviours. | Child Obesity Research Framework<br><br>Social Ecological Model<br><br>Social Cognitive Theory | Elementary schools (*n*=78)<br><br>Infrastructure schools (*n*=23)<br>non-infrastructure schools (*n*=21)<br>Comparison schools (*n*=34)<br><br>Texas | <u>Parent (*n*=2,053)</u><br><br><u>Student (*n*=3,315)</u><br>Girl<br>Infrastructure (47.5%)<br>Non-infrastructure (48.1%)<br>Comparison( 49.4%)<br><br>Boy<br>Infrastructure (52.5%)<br>Non-infrastructure (51.9%)<br>Comparison (50.6%)<br><br>White<br>Infrastructure (18.8%)<br>Non-infrastructure (25.7%)<br>Comparison (25.9%)<br><br>Black<br>Infrastructure (7.1%)<br>Non-infrastructure (7.0%)<br>Comparison (7.0%)<br><br>Latino or Hispanic<br>Infrastructure (70.3%)<br>Non-infrastructure | Not reported | Parent survey<br><br>Child survey | Descriptive analysis<br><br>Mixed linear regression modelling<br><br>Growth curve modelling | This study found that policies that provide cost-reimbursement funding for SR2S initiatives are able to achieve modest outcomes in the short term, but few differences are seen between providing larger amounts of funding for infrastructure projects compared with smaller amounts of funding for SR2S programs. |

| | | | | | | | | | | | | | | | |
|---|---|---|---|---|---|---|---|---|---|---|---|---|---|---|---|
| | | | | | | | | | | | (63.6%)<br>Comparison (63.5%)<br><br>Other<br>Infrastructure (3.8%)<br>Non-infrastructure (3.7%)<br>Comparison (3.6%) | | | | |
| [70] | Delaware Center for Transportation | 2011 | Grey Literature Report | Mixed method | Intervention | SR2S | USA | To provide a baseline active travel rate for the state and each county through analysis of survey data collected from parents of school-aged children living within walking distance of school and to understand why parents would either allow or not allow their children to walk or bicycle to school. | Not reported | Counties (*n*=3)<br><br>New Castle<br><br>Kent<br><br>Sussex<br><br>Delaware | <u>Quantitative</u><br>Parents (*n*= not reported)<br><br><u>Qualitative</u><br>Parents (*n*= not reported)<br><br>District administration (*n*= not reported)<br><br>Transport supervisor (*n*= not reported)<br><br>Community planner (*n*= not reported)<br><br>Health/walkability advocates (*n*= not reported) | <u>Quantitative</u><br>Not reported<br><br><u>Qualitative</u><br>Not reported | <u>Quantitative</u><br>Survey<br><br><u>Qualitative</u><br>Survey open-ended questions<br><br>Workshop | <u>Quantitative</u><br>Descriptive analysis<br><br><u>Qualitative</u><br>Thematic analysis | This study found that it is difficult to pin down the cultural, physical, demographic, and programmatic differences among the counties, which may well affect modal choice. The apparent divide between the provision of physical infrastructure and connectivity and the less-clear-cut obstacle of how to address or mitigate parental fear or disdain of healthy modal choices is clearly indicated. While the standard SR2S protocol calls for student polling regarding mode choice at participating schools, it lacks an insight into causality. |
| [71] | Mineta Transportation Institute | 2020 | Grey Literature Report | Mixed method | Intervention | SR2S | USA | To determine the relationship between Safe Routes to School programs and the likelihood that children will travel to school by active modes. | Theory of Routine Mode Choice Decisions | Counties (*n*=4)<br><br>Alameda<br>Contra Costa<br>San Mateo<br>Santa Clara County<br><br>San Francisco Bay Area<br><br>California | <u>Quantitative</u><br>Census tracts (*n*=965)<br><br><u>Qualitative</u><br>Administrators (*n*=3)<br><br>Parents (*n*=2) | <u>Household income</u><br><$10,000 (3%)<br>$10,000 to $24,999 (9%)<br>$25,000 to $34,999 (10%)<br>$35,000 to $49,999 (7%)<br>$50,000 to $74,999 (9%)<br>$75,000 to $99,999 (9%)<br>$100,000 to $149,999 (17%)<br>$150,000 to $199,999 (16%)<br>$200,000 to $249,999 (8%)<br>≥$250,000 (11%) | <u>Quantitative</u><br>Secondary data set (California Household Travel Survey)<br><br><u>Qualitative</u><br>Semi-structured interviews (*n*=5) | <u>Quantitative</u><br>Differences in means<br><br>Logistic regression modelling<br><br><u>Qualitative</u><br>Not reported | This report found students with longer commutes to school are less likely to use active modes. In terms of ethnicity, Asian/Pacific Islander students are less likely than their white peers to commute by active modes. The presence of SR2S programs at the school seems to reduce or even eliminate race-based and distance-based barriers to commuting to school by active modes. Walking and bicycling could be promoted through the five elements outlined in Schneider's theory of routine mode choice decision: awareness and availability, basic safety and security, convenience and cost, enjoyment, and habit. The school staff and volunteers identified several effective SR2S program elements related to each of these five steps. |
| [72] | National Center for Safe Routes to School | 2012 | Grey Literature Report | Mixed method | Intervention | SR2S | USA | To understand whether and how high performing schools and reference schools differ in the ways they established their SR2S programs. | Social Ecological Model | Schools (*n*=6)<br><br>Elementary (*n*=4)<br>Middle (*n*=2)<br><br>High performing (*n*=3)<br>Reference (*n*=3)<br><br>Suburban (*n*=2)<br>Urban (*n*=2)<br>Rural (*n*=2)<br><br>Minnesota | <u>Schools (*n*=6)</u><br><u>Non-white students</u><br>Pair 1<br>High performance (81.7%)<br>Reference (53.6%)<br><br>Pair 2<br>High performance (39.1%)<br>Reference (3.7%)<br><br>Pair 3<br>High performance (24.6%)<br>Reference (20.8%)<br><br><u>SR2S Program Coordinator (*n*=7)</u> | <u>Free/reduced price lunch</u><br>Pair 1<br>High performance (88.3%)<br>Reference (61.4%)<br><br>Pair 2<br>High performance (12.3%)<br>Reference (8.6%)<br><br>Pair 3<br>High performance (44.2%)<br>Reference (29.7%) | <u>Quantitative</u><br>Secondary data set (national Centre for Safe Routes to School)<br><br><u>Qualitative</u><br>Semi-structured telephone interviews (*n*-7) | <u>Quantitative</u><br>Comparative analysis<br><br><u>Qualitative</u><br>Not reported | This report found that program leadership, SR2S activity frequency, supportive policies and parent group engagement play key roles in encouraging more students to walk and bicycle to/ from school. |
| [73] | Oregon Transportation Research and Education Consortium | 2011 | Grey Literature Report | Mixed method | Intervention | SR2S | USA | To investigate how parental attitudes and time constraints affect decisions about children's transportation modes to school and to develop and test two methods of program evaluation and information gathering around Safe Routes to School that are not typically used in current program evaluations. | Not reported | School (*n*=4)<br><br>Elementary (*n*=2)<br>Middle (*n*=2)<br><br>Portland<br><br>Oregon | <u>Quantitative</u><br>Parent<br>Female (*n*=118)<br>Male (*n*=29)<br>Missing (*n*=2)<br><br>White (*n*=130)<br>Black (*n*=4)<br>Hispanic (*n*=6)<br>Asian (*n*=12)<br>Other (*n*=3)<br><br>Single parent (*n*=21)<br>Two parent/one employed (*n*=47)<br>Two parents/two employed (*n*=77)<br><br><u>Qualitative</u><br>Parent (*n*=59)<br><br>Female (*n*=49)<br>Male (*n*=10)<br><br>18 – 29 years (*n*=4)<br>30 – 39 years (*n*=23)<br>40 – 49 years (*n*=28)<br>50 – 59 years (*n*=2)<br>60 years or older (*n*=1)<br>Missing/Refused (*n*=1)<br><br>White or Caucasian (*n*=35)<br>Black or African American (*n*=8)<br>Hispanic or Latino/Latina (*n*=1) | <u>School SES</u><br>Low (*n*=2)<br>High (*n*=2)<br><br><u>Free or reduced price lunch</u><br>School A (99.6%)<br>School B (59.9%)<br>School C (14.4%)<br>School D (18.4%)<br><br><u>Minority students</u><br>School A (85.1%)<br>School B (67.1%)<br>School C (15.9%)<br>School D (15.8%)<br><br><u>English language learners</u><br>School A (13.1%)<br>School B (20.4%)<br>School C (1.4%)<br>School D (1.8%)<br><br><u>Quantitative</u><br><u>Parents education</u><br>Graduate school (*n*=70)<br>College degree (*n*=47)<br>Some college (*n*=23)<br>High school (*n*=8)<br><br><u>Qualitative</u><br>Not reported | <u>Quantitative</u><br>Parent survey<br><br>Child survey<br><br><u>Qualitative</u><br>Focus groups (*n*=4) | <u>Quantitative</u><br>Bivariate analysis<br><br>Multinomial analysis<br><br>Mixed logit modelling<br><br><u>Qualitative</u><br>Thematic analysis | This report found that the presence of a parent mentor or champion and specific encouragement programs, such as Walk and Bike to School Days, were most effective in influencing transportation choices. Distance and convenience were revealed to be primary factors in commute mode decisions, parents often use multiple travel modes within a day, a week or over the course of a year. |

| | | | | | | | | | | | | | | | |
|---|---|---|---|---|---|---|---|---|---|---|---|---|---|---|---|
| | | | | | | | | | | | American Indian or Alaskan Native (*n*=1)<br>Native Hawaiian or Pacific Islander (*n*=0)<br>Asian (*n*=0)<br>Other (*n*=0)<br>Missing/Refused (*n*=15)<br><br>Younger than Kindergarten (*n*=23)<br>Kindergarten through 5th Grade (*n*=0)<br>Kindergarten (*n*=10)<br>1st Grade (*n*=14)<br>2nd Grade (*n*=10)<br>3rd Grade (*n*=8)<br>4th Grade (*n*=5)<br>5th Grade (*n*=5)<br>6th Grade (*n*=3)<br>7th Grade (*n*=1)<br>8th Grade (*n*=1)<br>9th through 12th Grade (*n*=1)<br>Missing/Refused (*n*=1)<br><br>Attending Neighbourhood School<br>Yes (*n*=46)<br>No (*n*=13<br>Missing/Refused (*n*=0)<br><br>Length of Time in Current Neighbourhood<br>0.5 – 1.0 year (*n*=3)<br>1.5 – 3.0 years (*n*=6)<br>3.5 – 5.0 years (*n*=11)<br>6.0 – 8.0 years (*n*=14)<br>9.0 – 11.0 years (*n*=10)<br>12.0 – 18.0 years (*n*=8)<br>26.0 – 40.0 years (*n*=3)<br>Missing/Refused (*n*=4) | | | | |
| [74] | Safe Routes to School National Partnership | 2015 | Commentary | N/A | Intervention | SR2S | USA | To examine ways in which Safe Routes to School and community safety efforts overlap/complement each other and describe approaches to support personal safety for children and teens during the trip to and from school and broader community strategies. | Broken Windows Theory | N/A | N/A | N/A | N/A | N/A | This report found Violence and crime – and the fear of those events – restrict people's access to physical activity. Both the immediate effects of crime and violence, and the restrictions they impose on walking, bicycling, participation in outdoor activities, and connections with neighbours, can have grave effects on the physical and mental health of individuals and whole communities. While there is no panacea to address the challenges faced by communities with high crime, Safe Routes to School efforts can make one time of day tangibly safer, protecting children and youth from violence on their way to and from school. At the same time, Safe Routes to School proponents can become key conveners, contribute to comprehensive violence prevention approaches in communities, and assist in work on primary and secondary violence prevention approaches. Communities can strengthen violence prevention efforts by including Safe Routes to School approaches, with new tools, financial resources, discrete yet meaningful volunteer opportunities with children, and the social cohesion, health, and safety benefits of neighbours looking out for children. |
| [75] | Safe Transportation Research and Education Center | 2010 | Grey Literature Report | Mixed method | Intervention | SR2S | USA | To evaluate the WSB initiative across 10 low-income schools to identify lessons and recommendations for future programs. | Not reported | Elementary school (*n*=10)<br><br>Public (*n*=8)<br>Charter (*n*=2)<br><br>Urban (*n*=5)<br>Suburban (*n*=4)<br>Rural (*n*=1)<br><br>States (*n*=10)<br><br>California. Washington DC, Georgia, Illinois, Kentucky, Louisiana, New York, Oklahoma, Texas, Virginia | <u>Quantitative</u><br>Parents (*n*= 1,136)<br><br>Classroom (*n*= 14)<br><br><u>Qualitative</u><br>Caregivers (*n*=not reported)<br><br>School coordinator (*n*=10) | <u>Free/reduced price lunch</u><br>School A (93%)<br>School B (50%)<br>School C (96%)<br>School D(86%)<br>School E (77%)<br>School F (96%)<br>School G (77%)<br>School H (72%)<br>School I (41%)<br>School J (76%)<br><br><u>Quantitative</u><br>Not reported<br><br><u>Qualitative</u><br>Not reported | <u>Quantitative</u><br>Parent survey<br><br>Student tally<br><br>Traffic safety observation<br><br>Vehicle count<br><br><u>Qualitative</u><br>Semi-structured telephone interviews (*n*= not reported)<br><br>Focus groups (*n*= not reported) | <u>Quantitative</u><br>Descriptive analysis<br><br>Risk analysis<br><br><u>Qualitative</u><br>Not reported | This report found the project was successful and showed great promise for generating a continuing positive impact on health in the future. SR2S programs are not meant to be short-term. They must be ongoing and require continued investment. Long-term, continuing support through policy and programming has been shown to help promote physical activity among children. |
| [76] | Hawley *et al.* | 2019 | Research Article | Qualitative | Non-intervention | N/A | NZ | To explore the contextual factors associated with the success of the School Travel Planning program in exemplar schools. | Social Ecological Framework | Schools (*n*=9)<br><br>Primary (*n*=3)<br>Middle (*n*=4)<br>Secondary (*n*=2)<br><br>City school (*n*=5)<br>Provincial towns | School principle/representatives (*n*=9)<br><br>Students (*n*=7)<br>Girls (*n*=4)<br>Boys (*n*=3) | <u>School SES</u><br>High (*n*=5)<br>Medium (*n*=3)<br>Low (*n*=1) | Semi-structured interviews (*n*=9)<br><br>Focus groups (*n*=1) | Thematic analysis | This study found that a recursive relationship between the school culture, community culture and the physical environment exists within all exemplar schools. However, there was variation in the amount these factors influence each |

| | | | | | | | | | | | | | | | |
|---|---|---|---|---|---|---|---|---|---|---|---|---|---|---|---|
| | | | | | | | | | | school ($n$=4)<br><br>Urban ($n$=9)<br><br>North island ($n$=6)<br>South island ($n$=3) | 11-13 years ($n$=7) | | | | school, highlighting the unique nature of each school's context. |
| [77] | Sweeney & Von Hagen | 2016 | Research Article | Qualitative | Non-intervention | N/A | USA | To identify parent and child perceptions of the built and social environment, how these perceptions were formed, and how they influenced travel mode choice. | Not reported | Middle schools ($n$=3)<br><br>Communities ($n$=3)<br><br>New Jersey | Parents/guardians ($n$=48)<br><br>Female ($n$=37)<br>Male ($n$=11)<br><br>Child grade 6 ($n$=8)<br>Child grade 7 ($n$=16)<br>Child grade 8 ($n$=24) | Free/reduced price lunch<br>School A (32%)<br>School B (15%,)<br>School C (41%)<br><br>Median household income<br>School A ($78,821)<br>School B (78,625)<br>School C (89,992) | Semi-structed interviews ($n$=48) | Comparative content analysis<br><br>Thematic analysis | This study found differences in adult versus child perceptions and the emergence of several themes related to the environment and children's capacity for independence. Themes included differences in comfort with solo travel, which depended on time of day,<br>parental concern with abductions and sexual offenders, common use of cell phones and GPS technology to address safety fears, and perceptions with respect to the safety of travel modes on the basis of gender. |
| [78] | Clark & Dumas | 2020 | Research Article | Qualitative | Non-intervention | N/A | CAN | To draw on the Foucault-inspired notion of bio pedagogies to examine and problematise the salient factors of contemporary motherhood impacting children's active outdoor play (AOP). | The Foucauldian Framework | Urban and suburban neighbourhoods ($n$=not reported)<br><br>Gatineau and Ottawa | Parents ($n$=21)<br><br>Female ($n$=21)<br><br>26-35 ($n$=3)<br>36-45 ($n$=16)<br>46-55 ($n$=2)<br><br>French speaker ($n$=12)<br>English speaker ($n$=9) | Parent education<br>High school diploma ($n$=2)<br>Post-secondary ($n$=19) | Semi-structed interviews ($n$=21) | Thematic analysis | This study identified three discursive constructions of the 'good mother': mothers as time managers, risk managers and screen-time managers. Each was characterised by discursive conflicts that compromised the provision of AOP by mothers. |
| [79] | McDonald & Aalborg | 2009 | Research Article | Quantitative | Intervention | SR2S | USA | To understand why many parents choose to drive their children even short distances to school, and what implications this has for programs to increase walking and biking to school. | Not reported | Neighbourhoods ($n$=9)<br><br>Oakland, Berkeley, Albany, and Richmond<br><br>San Francisco Bay area<br><br>California | Parents ($n$=403)<br>Child's gender<br>Male (49.7%)<br>Female (50.3%)<br><br>Child's age<br>≤10 (19.3%)<br>11 (20.1%)<br>12 (20.3%)<br>13 (20.1%)<br>≥14 (20.3%)<br><br>Child's race<br>Non-Hispanic White (29.2%)<br>Non-Hispanic Black (15.2%)<br>Non-Hispanic Asian/ Pacific Islander (10.2%)<br>Hispanic (32.7%)<br>Multiracial/other (12.7%)<br><br>Distance to school<br>≤1 mile (41.7%)<br>1–2 miles (27.2%)<br>2–3 miles (11.0%)<br>>3 miles (20.2%) | Household income<br><$40,000 (25.8%)<br>$40,000 - $80,000 (36.6%)<br>> $80,000 (37.6%) | Telephone survey | Descriptive analysis | This study found to increase rates of walking to school SR2S program managers should offer non infrastructure programs that provide adult supervision and decrease the parental time costs of walking a child to school. We estimate a program addressing these concerns could affect the behaviour of 60% of parents who currently drive their children less than 2 miles to school. |
| [80] | Buckley *et al.* | 2013 | Research Article | Mixed method | Intervention | SR2S | USA | To evaluate the Safe Routes to School program's designated days for walking and bicycling in Moscow, Idaho. | Not reported | Elementary schools ($n$=3)<br><br>Intervention schools ($n$=2)<br>Control school ($n$=1)<br><br>Moscow<br><br>Idaho | Quantitative<br>Children ($n$=45)<br>Parents ($n$=17)<br><br>Qualitative<br>Parents ($n$=5)<br>Community leaders ($n$=4) | Not reported | Quantitative<br>Observational count data<br><br>Survey questionnaire<br><br>Qualitative<br>Telephone interviews ($n$=9) | Quantitative<br>Descriptive analysis<br><br>Qualitative<br>Not reported | This study found significant increase in students using active modes of travel on the day of the event and a few weeks later. There is strong support for the special events and most parents felt the designated days increased their child's motivation to walk to school. Many parents said the spring event prompted their child to return to walking to school after the cold winter months. |
| [81] | Zuniga | 2012 | Research Article | Qualitative | Non-intervention | N/A | USA | To examine how parents respond to barriers when they are encountered as part of the school journey. | Not reported | Elementary schools ($n$=12)<br><br>Denver<br><br>Colorado | Parents ($n$=65) | Not reported | Semi-structed interviews ($n$=64) | Content analysis | This study found two main themes associated parental response to AST (Barrier elimination and barrier negotiation). Regular use of AST reduces parental perception of barriers and introduces a phase of negotiation. Interventions should be designed to build capacity and inclination if they are going to influence active AST decision-making. |
